# IL-6 and cfDNA monitoring throughout COVID-19 hospitalization are accurate markers of its outcomes

**DOI:** 10.1186/s12931-023-02426-1

**Published:** 2023-05-05

**Authors:** Salvador Bello, Ana Belén Lasierra, Lucía López-Vergara, Cristina de Diego, Laura Torralba, Pablo Ruiz de Gopegui, Raquel Lahoz, Claudia Abadía, Javier Godino, Alberto Cebollada, Beatriz Jimeno, Carlota Bello, Antonio Tejada, Antoni Torres

**Affiliations:** 1 Department of Pulmonary Medicine, Miguel Servet University Hospital, CIBERES, Instituto de Investigación Sanitaria (ISS) Aragón, Avenida Isabel La Católica 1-9, 50009 Zaragoza, Spain; 2 Department of Biochemistry, San Jorge Hospital, Huesca, Spain; 3 Intensive Care Unit, Miguel Servet University Hospital, Zaragoza, Spain; 4 Department of Biochemistry, Miguel Servet University Hospital, Zaragoza, Spain; 5 Department of Cytometry and Cell Separation, Aragon Institute of Health Sciences (IACS), Zaragoza, Spain; 6 Biocomputing Technical Scientific Service, Aragon Institute of Health Sciences (IACS), Zaragoza, Spain; 7 Department of Radiology, Hospital Clínico Lozano Blesa, Zaragoza, Spain; 8 Servei de Pneumologia, Hospital Clinic, Universitat de Barcelona, IDIBAPS, ICREA, CIBERESUCICOVID, Barcelona, Spain

**Keywords:** COVID-19, Cell free DNA, IL-6, Immunity, Longitudinal study, Mortality, Severity

## Abstract

**Background:**

Severe COVID-19 entails a dysregulated immune response, most likely inflammation related to a lack of virus control. A better understanding of immune toxicity, immunosuppression balance, and COVID-19 assessments could help determine whether different clinical presentations are driven by specific types of immune responses. The progression of the immune response and tissular damage could predict outcomes and may help in the management of patients.

**Methods:**

We collected 201 serum samples from 93 hospitalised patients classified as moderately, severely, and critically ill. We differentiated the viral, early inflammatory, and late inflammatory phases and included 72 patients with 180 samples in separate stages for longitudinal study and 55 controls. We studied selected cytokines, P-selectin, and the tissue damage markers lactate dehydrogenase (LDH) and cell-free DNA (cfDNA).

**Results:**

TNF-α, IL-6, IL-8, and G-CSF were associated with severity and mortality, but only IL-6 increased since admission in the critical patients and non-survivors, correlating with damage markers. The lack of a significant decrease in IL-6 levels in the critical patients and non-survivors in the early inflammatory phase (a decreased presence in the other patients) suggests that these patients did not achieve viral control on days 10–16. For all patients, lactate dehydrogenase and cfDNA levels increased with severity, and cfDNA levels increased in the non-survivors from the first sample (p = 0.002) to the late inflammatory phase (p = 0.031). In the multivariate study, cfDNA was an independent risk factor for mortality and ICU admission.

**Conclusions:**

The distinct progression of IL-6 levels in the course of the disease, especially on days 10–16, was a good marker of progression to critical status and mortality and could guide the start of IL-6 blockade. cfDNA was an accurate marker of severity and mortality from admission and throughout COVID-19 progression.

**Supplementary Information:**

The online version contains supplementary material available at 10.1186/s12931-023-02426-1.

## Background

Coronavirus disease 2019 (COVID-19) can affect multiple organs, especially when severe, and its pathogenesis includes a dysregulated macrophage, neutrophil, and T and B-cell response, a proinflammatory cytokine release, and a cytopathic response from the virus, inducing progressive systemic inflammation, high neutrophil counts, and low lymphocyte counts [1]. Inflammatory response markers, such as leukocyte, neutrophil, and lymphocyte counts and C-reactive protein (CRP), ferritin, and D-dimer levels are used for managing hospitalized patients.

Markers of inflammatory response, such as leukocyte, neutrophil, and lymphocyte counts and CRP, ferritin, D-dimer, and interleukin (IL)-6 levels, have been widely employed for the management and decision-making process for hospitalized patients. COVID-19 patients show high levels of certain interleukins, endothelial grow factors, and other proinflammatory chemokines, as well as signaling proteins in serum [2]. The controversial cytokine storm in severe disease [3–5] has, along with other complications (especially lymphopenia), been associated with an elevated risk of acute respiratory distress syndrome and multiple organ failure [6]. T-cell exhaustion due to viral persistence, together with the impaired action of interferons produced by the virus, results in immunodepression and viral control failure, with additional inflammatory responses by damage-associated molecular patterns (DAMPs) released from damaged tissue [7]. Longitudinal studies during disease progression can be helpful in better understanding the immune toxicity and immunosuppression balance [8] especially when immune response inhibitors are a therapeutic option.

Since the beginning of the pandemic, the disease progression time has been considered important for prognostic and therapeutic measures. An approximately 7-day period has been theorized from symptom onset, characterized by the presence and shedding of virus (viral phase), followed by decreased shedding and increased inflammation (early inflammatory phase), in which the disease tends to worsen. If the inflammation and unbalanced immune response progress beyond 16 days (late inflammatory phase), severe lung/systemic disorders leading to critical disease and death can occur [9].

We conducted an observational, longitudinal, prospective study of a cohort of hospitalized patients with varying severity during the first COVID-19 pandemic wave, as well as controls. We examined clinical data, immune cell counts, proinflammatory cytokine levels, inflammatory markers, and tissue damage-related molecules to determine their role in predicting outcomes and improving patient management. Our hypotheses were that (a) different immune responses correlate with differing degrees of severity and that (b) the levels of certain components of the immune response and of tissular damage throughout the course of the disease can be markers of favorable or unfavorable outcomes and could guide the use of certain therapies.

## Methods

### Patients and controls

In April and May 2020, symptomatic patients hospitalized with COVID-19 demonstrated by polymerase chain reaction or nasopharyngeal swab were recruited. The exclusion criteria were concomitant infection on admission or during hospitalization, refusal to sign the informed consent, and significant immunosuppression (transplant recipients, hematologic neoplasms, chemotherapy, prednisone equivalent ≥ 20 mg/day). Based on their Chinese Center for Disease Control and Prevention classification (CCDC score) [10] and the World Health Organization Ordinal Scale (WHO OS) [11], the patients were grouped according to severity as follows: moderately ill (MI), severely ill (SI), and critically ill (CI).

After recording the date of symptom onset on admission to the hospital, we considered 3 phases: viral (1–9 days from symptom onset), inflammatory (10–16 days from symptom onset), and late inflammatory (> 16 days from symptom onset). The samples were obtained from blood requested for healthcare reasons. Patients with at least 2 blood samples in 2 consecutive phases were included in the longitudinal study.

We measured proinflammatory cytokines from the early innate immune response (tumor necrosis factor alpha [TNF-α], IL-1β, IL-6), related to neutrophil activation and recruitment (IL-8, granulocyte colony-stimulating factor [G-CSF]), and with T-cell response (IL-17A, interferon gamma [IFN-γ]), endothelial damage marker P-selectin, and tissue damage markers lactate dehydrogenase (LDH) and cell-free DNA (cfDNA).

We selected 55 non-hospitalized controls with no infection, of similar age and chronic medical conditions as the patients. All of the controls underwent routine laboratory tests and met the same exclusion criteria as the patient group. To compare the three dependent patient groups (severity groups and the three disease progression phases), we would need a total sample size of 100 (33 per group).

### Blood samples

Blood was collected in anticoagulant ethylenediaminetetraacetic acid tubes and immediately subjected to hematological analysis (complete blood count and D-dimer levels). Blood was also collected in serum separator tubes containing clot activator and serum separator gel (BD Vacutainer, BD Vacutainer SST II Advance; BD Frankin Lakes, NJ, USA). Serum samples were analyzed and included the following biomarkers: ferritin, LDH (Kinetics [340 nm]/Lactate to Pyruvate, AU Beckman), IL-6 (ECLIA Cobas-Roche), CRP (Latex particle immunoturbidimetry, AU Beckman), and procalcitonin (ECLIA Cobas-Roche). The remaining serum was stored at 4 °C for up to 24 h before separation into small aliquots and stored at − 80 °C until testing for cytokines, P-selectin, and cfDNA.

### Cytokines and P-selectin

Serum cytokines (except for IL-6) and P-selectin were quantified using a bead-based multiplex immunoassay (LXSAHM-07) according to the manufacturer’s instructions. Cytokines were quantified using a LABScan 100 flow analyzer (Magnetic Luminex Assay 7 Plex LXSAHM-07, BIO-TECHNE R&D systems, Minneapolis, MN, USA). Data were analyzed with xPONENT software.

### Cell-free DNA

To quantify the circulating serum cfDNA, DNA was first purified using the ChargeSwitch gDNA 1 mL Serum Kit (ChargeSwitch gDNA 1 mL Serum kit CS11040, Invitrogen, ThermoFisher Scientific Waltham, MA, USA), following the manufacturer’s procedure, using 100 μL of sample and adjusting the reactant quantity to that volume. After purification, the DNA was quantified using the Quant-iT PicoGreen dsDNA Assay Kit (Invitrogen P7589). After diluting the sample to 1/5 in TE buffer, PicoGreen reagent was added in a 1:1 ratio. After a 5-min incubation at room temperature in the dark, the sample’s fluorescence was measured using a Synergy HT reader (BioTek) (excitation 485 nm, emission 528 nm). cfDNA concentrations were calculated using Microsoft Excel with the standard provided in the kit.

### Statistical analysis

Data are presented as mean (standard deviation) or median (interquartile range), according to normal criteria (Shapiro-Wilks test) and analyzed with Mann–Whitney U, Kruskal–Wallis, Student’s or ANOVA tests, as appropriate. Pearson’s correlation coefficient was used to assess the relationship between biomarkers and clinical variables. The various biomarkers’ predictive power for different outcomes was analyzed using receiver operating characteristic (ROC) analysis.

For the longitudinal study (72 patients, 180 samples), a Wilcoxon signed-rank test was performed. Univariate/multivariate logistic regression models were performed for specific outcomes, adjusting for an age > 60 years, male sex, hypertension, obesity, diabetes, IL-6 levels, and corticosteroid therapy. p-values < 0.05 were considered statistically significant using R version 4.0.5 (R Core Team 2021) software.

## Results

### Patients and controls

We collected 201 samples from 93 patients, 72 of whom had samples (180) in at least 2 disease progression stages. Table 1 shows the distribution on the day the samples were extracted. Twelve of the patients required admission to the intensive care unit (ICU) and 19 died.

**Table 1:** Distribution of the day of sample extraction

	Viral phase (1–9 days) N = 87	Early inflammatory (10–16 days) N = 53	Late inflammatory (> 16 days) N = 40
Days from symptom onset	6.00 [5.00; 8.00]	12.00 [11.00; 14.00]	20.00 [18.00; 24.25]

Of the 180 samples from the 72 patients, 87 corresponded to the viral phase, 53 to the early inflammatory phase and 40 to the late inflammatory phase. Thirty-two patients had three or more samples, and eight patients had samples from all three disease progression phases.

Table 2 presents the patients’ information according to severity and samples at each stage. There were no statistically significant differences in the demographics and comorbidities of the patients and controls (Table 3). At admission, 56 of the study 93 patients had moderate disease, 29 had severe disease, and 8 were initially classified as critical.

**Table 2:** **A** Distribution of the 93 patients included in the study according to the maximum degree of severity reached; **B** Distribution of the samples obtained in the three evolutionary phases, according to time from symptom onset, of the total number of patients (93 patients, 201 samples) and of the patients followed longitudinally, with at least two samples in two consecutive evolutionary phases (72 patients, 180 samples)

A				
CCDC scale	Mild-moderate	Severe	Critical	Total
Patients (n)	51	15	27	93

WHO OS	1	2	3	4	5	6	7	8	Total
Patients (n)	0	0	7	41	20	1	5	19	93

B				
Illness onset	1–9 days	10–16 days	≥ 17 days	Total
93 patient samples (n)	97	59	45	201
72 patient samples (n)	87	53	40	180

WHO Ordinal Scale: 1–2: Mild; 3–4: Moderate; 5–6: Severe; 7: Critical; 8: Dead

*CCDC* Chinese Center for Disease Control and Prevention classification, *WHO OS* World Health Organization Ordinal Scale

**Table 3:** Demographics and comorbidities of the cases and controls

		Patients N = 93	Controls N = 55	*p*
Sex	Female	51 (54.84%)	30 (54.55%)	1.000
	Male	42 (45.27%)	25 (45.45%)	
Age (years)		69.00 [55.00; 78.00]	71.00 [57.00; 79.00]	0.441
Hypertension		45 (48.39%)	17 (30.91%)	0.056
Diabetes		28 (30.11%)	12 (21.82%)	0.365
Obesity		13 (13.98%)	4 (7.27%)	0.332
Heart disease		14 (15.05%)	6 (10.91%)	0.643
Chronic respiratory disease		16 (17.20%)	3 (5.45%)	0.070
Liver disease		3 (3.23%)	0 (0.00%)	0.295
Renal disease		8 (8.60%)	6 (10.91%)	0.863
Dementia		18 (19.35%)	4 (7.27%)	0.079
Dyslipidemia		17 (18.28%)	17 (30.91%)	0.118

Advanced age, male sex, and obesity were associated with more severe inflammatory responses and tissue damage (See Additional file 2: Table S1). In the multivariate study, obesity was a risk factor for a critical condition (odds ratio [OR] 9.25, p = 0.019) and ICU admission (OR 46.21, p = 0.008) (See Additional file 3: Table S2). Lymphopenia was always related to severity/mortality and correlated with tissue damage markers. Lymphocyte count declined with mortality and as severity progressed (See Additional file 4: Table S3, Additional file 5: Table S4, Additional file 6: Table S5 and Additional file 7: Table S6).

We observed higher G-CSF, IL-6, and cfDNA levels in the patients than in the controls, starting with the first sample. However, there were no statistically significant differences in TNF-α, IL-1β, IL-8, and IL-17A levels in the patients and controls, and even higher levels were observed in the controls when comparing them with only the first patient sample (p < 0.05) (Table 4); however, all cytokine levels increased during disease progression. The Additional file 8: Figure S1 show a graphical representation of the correlations between the biomarkers in the 3 disease phases, as well as a comparison of biomarkers in the 3 severity groups (CCDC score) (See Additional file 9: Table S7).

**Table 4:** Comparison of biomarkers between patients and controls for all samples and for only the first sample after admission

All samples	Patients N = 201	Controls N = 55	p
TNF-α (pg/mL)	40.02 [34.83; 47.41]	41.07 [38.56; 44.99]	0.156
IL-8 (pg/mL)	54.29 [40.43; 89.22]	56.21 [43.26; 107.75]	0.412
IL-1β (pg/mL)	55.90 [47.80; 66.92]	59.91 [54.04; 62.24]	0.238
IFN-γ (pg/mL)	145.84 [123.72; 176.59]	134.60 [128.65; 142.43]	0.004
IL-17A (pg/mL)	19.40 [14.73; 23.90]	21.31 [18.48; 21.31]	0.204
P-Selectin (ng/mL)	55.48 [42.12; 73.30]	77.44 [67.55; 84.99]	< 0.001
G-CSF (pg/mL)	143.97 [123.70; 159.45]	130.47 [124.32; 138.97]	< 0.001
IL-6 (pg/mL)	20.41 [4.34; 51.62]	7.80 [5.15; 10.42]	0.004
cfDNA (ng/mL)	7.87 [4.45; 14.40]	2.56 [1.71; 3.61]	< 0.001

1st samples	Patients N = 93	Controls N = 55	p
TNF-α (pg/mL)	38.51 [32.58; 45.34]	41.07 [38.56; 44.99]	0.013
IL-8 (pg/mL)	47.45 [33.71; 66.16]	56.21 [43.26; 107.75]	0.010
IL-1β (pg/mL)	51.66 [43.09; 59.90]	59.91 [54.04; 62.24]	< 0.001
IFN-γ (pg/mL)	134.40 [119.97; 159.07]	134.60 [128.65; 142.43]	0.846
IL-17A (pg/mL)	18.48 [10.79; 22.80]	21.31 [18.48; 21.31]	0.001
P-Selectin (ng/mL)	52.21 [35.63; 71.42]	77.44 [67.55; 84.99]	< 0.001
G-CSF (pg/mL)	142.57 [120.38; 162.04]	130.47 [124.32; 138.97]	0.041
IL-6 (pg/mL)	30.52 [6.26; 62.51]	7.80 [5.15; 10.42]	< 0.001
cfDNA (ng/mL)	7.68 [4.23; 14.49]	2.56 [1.71; 3.61]	< 0.001

*TNF-α* tumor necrosis factor-α, *IL-8* interleukin-8, *IL-1β* interleukin-1β, *IFN-γ* interferon- γ, *IL-17A* interleukin 17A, *G-CSF* Granulocyte colony-stimulating factor, *IL-6* interleukin-6, *cfDNA* cell free DNA

### Cytokines and tissue damage markers

**Fig. 1:**
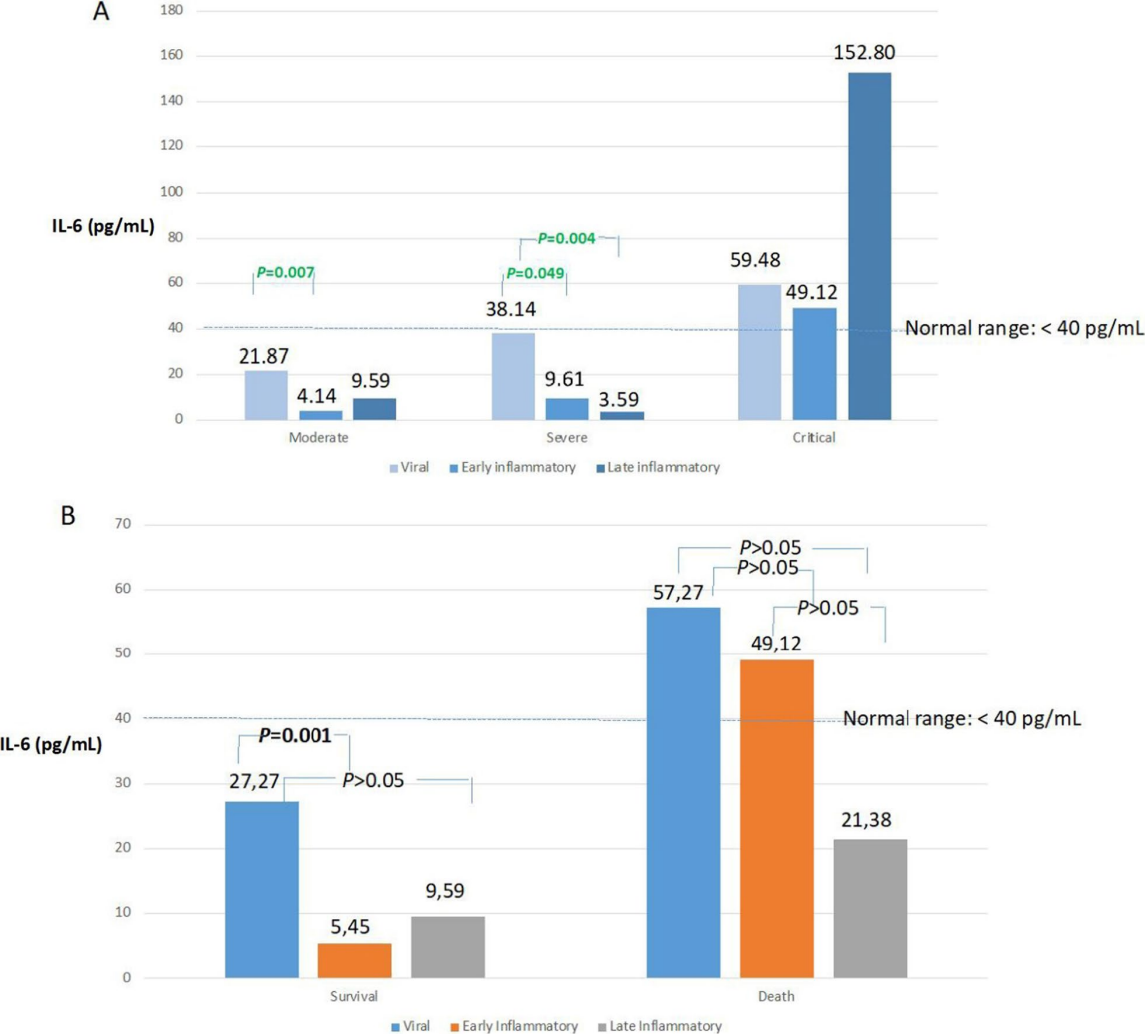
Median interleukin-6 levels through COVID-19 progression according to (**A**) severity (World Health Organization Ordinal Scale) and (**B**) mortality

There were higher levels of cfDNA, TNF-α, IL-8, G-CSF, and especially IL-6 in the CI group (as measured by both scales) and non-survivors (Table 5). The median IL-6 values did not exceed the limit of 40 pg/mL at any time during disease progression for the MI, SI, and survivor groups, while the values remained above this limit for the CI and non-survivor groups (Fig. 1). IL-6 was the only cytokine studied that correlated with the tissue damage markers LDH and cfDNA. The Additional file 4: Table S3, show the relationship between the biomarkers and respiratory severity parameters.

**Table 5:** Comparisons of interleukin-6 and cfDNA levels according to severity and between survivors and non-survivors

		Severe and Critical	Moderate	p
CCDC scale	IL-6 (pg/mL)	32.44 [5.84; 104.03]	9.00 [2.76; 33.84]	< 0.001
	cfDNA (ng/mL)	9.44 [6.15; 20.19]	6.21 [3.46; 10.44]	< 0.001
WHO OS	IL-6 (pg/mL)	32.44 [5.69; 99.94]	9.00 [2.90; 33.14]	< 0.001
	cfDNA (ng/mL)	9.08 [5.54; 19.38]	6.88 [3.61; 10.73]	0.001

		Critical	Moderate and Severe	p
CCDC scale	IL-6 (pg/mL)	59.48 [21.37; 175.20]	10.06 [3.10; 34.21]	< 0.001
	cfDNA (ng/mL)	14.11 [7.13; 22.67]	7.01 [4.01; 10.93]	< 0.001

		Survivors	Non-survivors	p
Total samples (n = 201)	IL-6 (pg/mL)	11.14 [3.49; 39.23] n = 158	52.49 [21.37; 152.90] n = 43	< 0.001
	cfDNA (ng/mL)	7.06 [4.12; 11.40] n = 158	14.98 [7.42; 25.74] n = 43	< 0.001
1st sample (n = 93)	IL-6 (pg/mL)	25.91 [5.77; 51.55] n = 74	66.91 [38.38; 162.70] n = 19	0.001
	cfDNA (ng/mL)	6.87 [4.01; 10.81] n = 74	14.98 [7.73; 22.51] n = 19	0.002

*IL-6* interleukin-6, *cfDNA* cell-free DNA, *CCDC* Chinese Center for Disease Control and Prevention classification, *WHO OS* World Health Organization Ordinal Scale

In our longitudinal study, we observed a statistically significant decrease (p = 0.021) in IL-6 levels from days 10–16 (10.38 [2.59; 31.52]) versus days 1–9 (29.11 [6.49; 58.41]) (Table 6). A similar statistically significant decrease (p = 0.001) in IL-6 levels was observed on days 10–16 (5.28 [1.50; 23.10]) versus days 1–9 (27.27 [5.78; 51.08]) in the survivors but not in the non-survivors (Fig. 1 and Additional file 10: Table S8). We also analyzed the IL-6 levels through the COVID-19 progression phases according to severity: per the CCDC score, there was a sevenfold and threefold decrease in the MI (p = 0.006) and SI groups, respectively, for days 1–9 versus days 10–16. Per the WHO OS, there was a fivefold and fourfold decrease in the MI (p = 0.007) and SI (p = 0.049) groups, respectively (Fig. 1). This IL-6 decrease was maintained in the late inflammatory phase in the MI, SI, and survivor groups. In contrast, the CI group (WHO OS) presented a clear increase in IL-6 in the late inflammatory phase (152.8 [12.88; 447.90]). In the SI group, the statistically significant IL-6 decrease in the early inflammatory phase coincided with normalization of their initial lymphopenia, in contrast to the CI group.

**Table 6:** Comparison of biomarkers during the three disease progression phases

	Viral phase (1) N = 87	Early inflammatory (2) N = 53	Late inflammatory (3) N = 40	p	p 1 vs 2	p 1 vs 3	p 2 vs 3
Leukocytes/mm^3^	7000 [5300;9050]	8000 [6400;11400]	8200 [6450;11800]	0.034	0.092	0.056	0.582
Neutrophils/mm^3^	5100 [3350;6950]	6000 [4400;8700]	6050 [4100;8225]	0.069	0.119	0.119	0.874
Lymphocytes/mm^3^	1000 [650;1400]	1000 [700;1600]	1150 [675;1900]	0.705	0.858	0.858	0.858
N/L ratio	5.12 [2.71;9.11]	6.02 [2.61;14.35]	4.96 [2.74;11.03]	0.700	0.836	0.836	0.836
Platelet/mm^3^	234,000 [167500;285500]	278,000 [197000;367000]	227,000 [186000;278250]	0.030	0.028	0.667	0.114
CRP (mg/L)	50.00 [17.70;108.05]	25.10 [9.40;74.60]	17.00 [3.08;51.45]	0.004	0.032	0.011	0.261
PCT (ng/mL)	0.08 [0.05;0.16]	0.07 [0.04;0.12]	0.08 [0.04;0.23]	0.415	0.564	0.620	0.620
LDH (U/L)	294.50 [223.25;385.25]	274.00 [201.00;345.00]	232.00 [173.00;327.25]	0.127	0.362	0.135	0.362
D-Dimer (µg/L)	865.00 [434.00;1464.00]	855.50 [495.25;1597.75]	698.50 [345.50;1951.25]	0.776	0.998	0.848	0.848
Ferritin (ng/mL)	445.00 [219.80;620.90]	647.50 [321.67;945.40]	397.00 [248.50;726.10]	0.107	0.103	0.808	0.343
TNF-α (pg/mL)	36.40 [32.80;43.59]	44.28 [38.81;49.81]	41.11 [37.36;48.52]	< 0.001	0.001	0.010	0.381
IL-8 (pg/mL)	52.01 [34.96;68.72]	55.52 [45.64;91.01]	77.32 [50.36;106.06]	0.002	0.048	0.002	0.108
IL-1β (pg/mL)	50.63 [43.09;61.88]	60.12 [54.10;69.95]	64.34 [53.61;69.23]	< 0.001	< 0.001	< 0.001	0.954
IFN-ʏ (pg/mL)	131.17 [116.20;158.99]	164.89 [143.24;179.26]	157.76 [134.66;188.20]	< 0.001	< 0.001	0.001	0.578
IL-17A (pg/mL)	18.48 [10.79;22.80]	21.69 [18.71;28.26]	23.90 [15.45;24.77]	< 0.001	< 0.001	0.001	0.714
P-Selectin (ng/mL)	47.42 [34.91;60.86]	65.42 [49.03;80.14]	64.95 [51.05;79.88]	< 0.001	< 0.001	< 0.001	0.842
G-CSF (pg/mL)	139.08 [120.98;160.64]	147.85 [129.84;162.19]	148.79 [132.43;157.64]	0.478	0.488	0.488	0.964
IL-6 (pg/mL)	29.11 [6.49;58.41]	10.38 [2.59;31.52]	10.06 [3.20;50.89]	0.024	0.021	0.190	0.552
cfDNA (ng/mL)	6.40 [3.61;13.99]	7.87 [5.34;14.65]	10.53 [5.56;17.30]	0.041	0.200	0.043	0.200
SaO_2_/FiO_2_	444.50 [365.00;460.75]	429.00 [339.00;457.00]	330.50 [170.75;467.75]	0.832	0.938	0.938	0.938
SaO_2_	94.00 [88.50;96.00]	90.00 [87.00;95.00]	93.00 [88.00;95.00]	0.035	0.057	0.109	0.466

*N/L ratio* neutrophyls/lymphocytes ratio, *CRP* C-reactive protein, *PCT* procalcitonin, *LDH* lactate dehydrogenase, *TNF-α* tumor necrosis factor-α, *IL-8* interleukin-8, *IL-1β* interleukin-1β, *IFN-γ* interferon- γ, *IL-17A* intereleukin-17A, *G-CSF* Granulocyte colony-stimulating factor, *IL-6* interleukin-6, *cfDNA* cell free DNA, *SaO*_*2*_*/ FiO*_*2*_ oxygen saturation/fraction of inspired oxygen, *SaO*_*2*_ oxygen saturation

Serum IL-1β, IFN-γ, and IL-17A levels had no relationship with severity. IL-8, G-CSF, IL-6, and cfDNA were associated with mortality from the first sample onwards (Table 7).

**Table 7:** Comparison of biomarkers between survivors and non-survivors in the first sample

	Survivors N = 74	Non-survivors N = 19	p
Leukocytes/mm^3^	7200 [5425;8775]	9800 [6600;13150]	0.028
Neutrophils/mm^3^	5050 [3725;6550]	8400 [4950;11750]	0.009
Lymphocytes/mm^3^	1200 [800;1600]	700 [532.50;800]	0.001
N/L ratio	4.46 [2.51;7.72]	13.50 [6.75;20.95]	0.001
Platelet/mm^3^	245,000 [168750;279750]	228,000 [192000;327000]	0.675
CRP (mg/L)	33.90 [11.70;95.10]	104.10 [59.20;197.00]	0.008
PCT (ng/mL)	0.07 [0.04;0.11]	0.14 [0.08;0.60]	0.025
LDH (U/L)	255.00 [208.50;366.50]	323.00 [237.50;403.50]	0.223
D-Dimer (µg/L)	828.50 [471.75;1378.50]	1533.50 [1009.75;2671.00]	0.009
Ferritin (ng/mL)	427.30 [176.00;623.90]	491.70 [392.35;963.60]	0.110
TNF-α (pg/mL)	37.84 [31.76;44.02]	40.91 [32.95;49.72]	0.194
IL-8 (pg/mL)	42.11 [33.18;56.48]	61.83 [52.88;89.06]	0.016
IL-1β (pg/mL)	50.63 [43.09;58.71]	53.75 [45.44;65.57]	0.314
IFN-ʏ (pg/mL)	132.22 [119.97;154.89]	144.93 [117.45;166.73]	0.737
IL-17A (pg/mL)	15.67 [10.79;22.80]	19.40 [13.35;22.53]	0.287
P-Selectin (ng/mL)	49.25 [35.63;71.35]	53.59 [36.85;69.489]	0.900
G-CSF (pg/mL)	134.22 [118.12;153.99]	160.09 [139.92;184.10]	0.012
IL-6 (pg/mL)	25.91 [5.77;51.55]	66.91 [38.36;162.70]	0.001
cfDNA (ng/mL)	6.87 [4.01;10.81]	14.98 [7.73;22.51]	0.002
SaO_2_/FiO_2_	452.00 [425.50;462.00]	380.00 [323.00;416.50]	0.003
SaO_2_ (%)	95.00 [90.00;97.00]	85.00 [75.50;86.50]	< 0.001

*N/L ratio* neutrophyls/lymphocytes ratio, *CRP* C-reactive protein, *PCT* procalcitonin, *LDH* lactate dehydrogenase, *TNF-α* tumor necrosis factor-α, *IL-8* interleukin-8, *IL-1β* interleukin-1β, *IFN-γ* interferon- γ, *IL-17A* intereleukin-17A, *G-CSF* Granulocyte colony-stimulating factor, *IL-6* interleukin-6, *cfDNA* cell free DNA, *SaO*_*2*_*/ FiO*_*2*_ oxygen saturation/fraction of inspired oxygen, *SaO*_*2*_ oxygen saturation

The MI group had a statistically significant increase in TNF-α, IL-8, IL-1β, IFN-γ, IL-17A, and P-selectin levels from the viral phase to the early/late inflammatory phase but not from the early to the late inflammatory phases. The SI group had stable levels while the CI group had decreased levels, unlike the situation with the IL-6 levels.

**Fig. 2:**
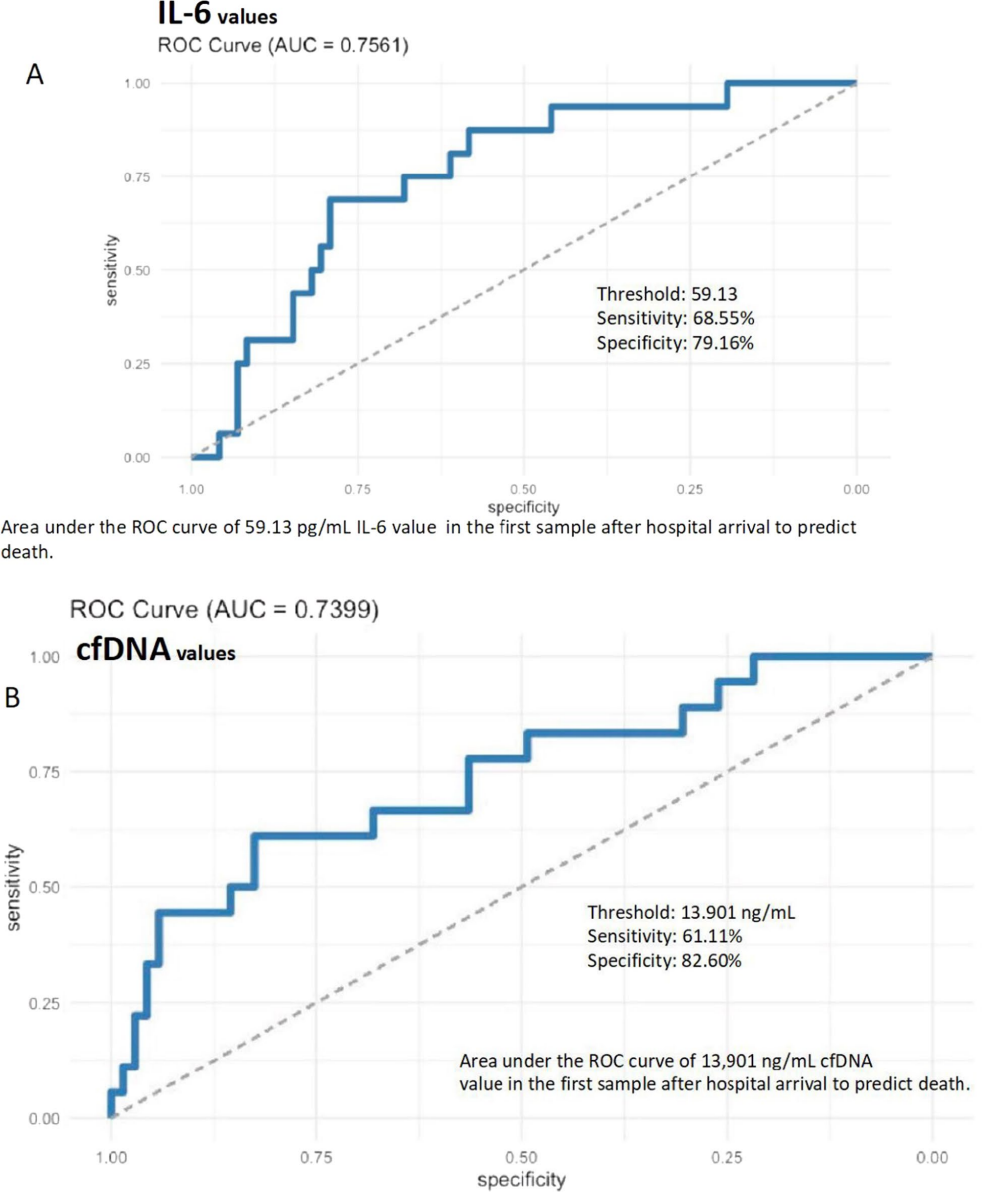
Area under the curve for mortality of (**A**) interleukin-6 and (**B**) cell-free DNA from the first sample after hospital admission

IL-6 levels correlated significantly with LDH throughout the 3 disease progression phases, in the 3 severity groups (Additional file 11: Table S9), and with cfDNA in the early inflammatory phase, showing significantly higher levels from the first sample onwards in the CI and non-survivor groups. In the ROC analysis, the area under the curve for an IL-6 concentration of 59.13 in the first sample for predicting death was 0.7561 (sensitivity 0.6875, specificity 0.7916) (Fig. 2).

LDH levels increased as severity increased; unlike IL-6 and cfDNA, however, LDH was not related to mortality in the first sample (Table 7) and showed higher levels only in the late phase of the non-survivors (p = 0.001). LDH, IL-6, CRP, and cfDNA levels increased in the patients with baseline SaO_2_ < 93%, SaO_2_/FiO_2_ < 315, and with radiological infiltrates in > 50% of the lung fields (Additional file 4: Table S3).cfDNA levels were significantly higher in the CI group than in the MI (p < 0.001) and SI groups (p = 0.004) (according to the CCDC score) and were higher in the SI and CI groups than in the MI group of WHO OS (p = 0.001) (Table 8) and non-survivors, starting with the first sample after admission (p = 0.002) (Table 5). Along with neutrophil count, lymphopenia, N/L ratio, and CRP, cfDNA was the only persistent and significantly higher biomarker in the non-survivors during the 3 disease phases (Table 9). In the late inflammatory phase, median cfDNA levels were quadruple the initial values (p = 0.031) in the non-survivors, remaining stable in the survivors (Table 8). In the CI group, cfDNA levels were double those of the MI and SI groups (Additional file 9: Table S7), during the entire disease progression and were correlated with hospital stay for the survivors (r = 0.311, p = 0.000).

**Table 8:** cfDNA according to severity (Chinese Center for Disease Control and World Health Organization scales) and mortality and its correlation with interleukin-6 and lactate dehydrogenase (N = samples)

Severity	cfDNA (ng/mL)	p
CCDC scale		< 0.001
Moderate (N = 105)	6.42 (3.49–10.48)	
Severe (N = 38)	7.47 (5.32–13.37)	
Critical (N = 58)	14.92 (7.18–24.68)	
Critical vs. moderate		< 0.001
Critical vs. severe		0.004
WHO OS		0.001
Moderate; N = 102	6.97 (3.76–10.83)	
Severe/Critical; N = 99	9.36 (5.56–19.60)	

Mortality	cfDNA (ng/mL)	p
Non-survivors N = 39		0.021
Viral (1–9 days); N = 17	7.87 [5.52–22.38]	
Early inflammatory (10–16 days); N = 11	12.59 [7.88–26.36]	
Late inflammatory (> 17 days); N = 11	30.34 [18.47–39.39]	
Viral phase *vs* late inflammatory		0.031
Survivors N = 141		0.328
Viral (1–9 days); N = 70	5.98 (3.12–11.45)	
Early inflammatory (10–16 days); N = 42	7.11 (4.59–10.60)	
Late inflammatory (> 17 days); N = 29	7.83 (4.65–12.18)	

Correlation cfDNA with IL-6 and LDH	r	p
IL-6		
Viral (1–9 days)	0.12, (− 0.104–0.332)	0.294
Early inflammatory (10–16 days)	0.468 (0.192–0.672)	0.002
Late inflammatory (> 17 days)	− 0.005 (− 0.348–0.339)	0.977
LDH		
Viral (1–9 days)	0.296 (0.079–0.486)	0.009
Early inflammatory (10–16 days)	0.376 (0.115–0.589)	0.006
Late inflammatory (> 17 days)	0.294 (− 0.024–0.558)	0.069

*cfDNA* cell-free DNA, *CCDz* Chinese Center for Disease Control and World Health Organization scale,* WHO OS* World Health Organization Ordinal Scale, *IL* interleukin, *LDH* lactate dehydrogenase

**Table 9:** Comparison of biomarkers between survivors and non-survivors in the 3 phases of the disease

Viral phase (1–9 days) N = 97	Survivors N = 78	Non-survivors N = 19	p
Neutrophils/mm^3^	4500 [3150;6600]	7000 [4600;10550]	0.011
Lymphocytes/mm^3^	1200 [800;1600]	600 [450;800]	< 0.001
N/L ratio	4.25 [2.10;7.49]	13.50 [6.75;25.85]	< 0.001
CRP (mg/L)	38.0 [12.33;93.83]	95.00 [59.20;153.40]	0.047
Ferritin (ng/mL)	406.00 [176.00;569.50]	558.90 [460.75;1046.17]	0.015
IL-6 (pg/mL)	27.27 [5.78;51.08]	57.27 [31.73;98.75]	0.039
cfDNA (ng/mL)	6.11 [3.48;11.45]	7.87 [5.08;22.21]	0.037

Early inflammatory (10–16 days) N = 59	Survivors N = 48	Non-survivors N = 11	p
Leukocytes/mm^3^	7500 [6475;8825]	12,600 [8900;16250]	0.004
Neutrophils/mm^3^	5600 [4275;7000]	11,400 [7650;14550]	< 0.001
Lymphocytes/mm^3^	1200 [775;1800]	700 [400;800]	0.002
N/L ratio	4.40 [2.39;9.06]	17.14 [15.59;24.93]	< 0.001
CRP (mg/L)	20.30 [5.65;41.20]	72.25 [38.75;128.12]	0.004
PCT (ng/mL)	0.05 [0.04;0.11]	0.12 [0.07;0.21]	0.023
D-Dimer (µg/L)	705.50 [469.50;1020.75]	3256.50 [1292.25;4987.75]	0.014
IL-6 (pg/mL)	5.28 [1.50;23.10]	49.12 [26.78;214.49]	0.001
cfDNA (ng/mL)	7.11 [4.46;9.75]	12.59 [7.88;26.36]	0.008

Late inflammatory (> 16 days) N = 45	Survivors N = 32	Non-survivors N = 13	p
Leukocytes/mm^3^	7400 [6300;10250]	9800 [8700;18700]	0.003
Neutrophils/mm^3^	5250 [4075;6625]	8400 [6500;16400]	0.001
Lymphocytes/mm^3^	1300 [800;2300]	600 [400;1100]	0.002
N/L ratio	3.82 [2.55;6.19]	15.50 [7.64;41.00]	< 0.001
CRP (mg/L)	9.40 [1.75;19.70]	236.50 [138.15;459.38]	< 0.001
PCT (ng/mL)	0.06 [0.04;0.09]	0.34 [0.26;0.88]	< 0.001
LDH (U/L)	199.00 [170.50;275.50]	341.50 [298.75;446.00]	0.001
D-Dimer (µg/L)	692.00 [336.50;1619.50]	2392.00 [1525.00;2925.50]	0.020
cfDNA (ng/mL)	8.35 [4.91;12.94]	24.62 [19.21;39.06]	< 0.001

*N/L ratio* neutrophyls/lymphocytes ratio, *CRP* C-reactive protein, *PCT* procalcitonin, *IL-6* interleukin-6, *cfDNA* cell free DNA, *LDH* lactate dehydrogenase

The correlation between cfDNA and LDH was not robust (Table 8), and cfDNA correlated better than LDH with common severity markers (neutrophilia, lymphopenia, CRP, SaO_2_, SaO_2_/FiO_2_), especially in the CI group and late inflammatory phases (Additional file 8: Figure S1). The ROC analysis showed a mortality area under the curve of 0.7399 for cfDNA in the first sample (sensitivity 0.6111, specificity 0.8260) (Fig. 2).cfDNA was the only biomarker that was an independent risk factor for ICU admittance (OR 1.22, p = 0.025) and mortality (OR 1.08, p = 0.014) (Additional file 3: Table S2).

### Therapies

Of the 93 patients, only 6 received tocilizumab, 4 received remdesivir, and 29 received corticosteroids, the latter of whom showed a higher degree of severity according to the CCDC (p = 0.025) and WHO (p = 0.008) scales but were not associated with lower in-hospital mortality (p > 0.05). Samples obtained in the early viral (and inflammatory phase) of those treated with corticosteroids showed clinical and analytical parameters of greater severity than those not treated (Additional file 12: Table S10), while no differences were observed after day 17 of progression between the two groups. In the multivariate model, taking corticosteroids was an independent risk factor for pneumonia (OR 4.55, p = 0.034) and extension of infiltrates in > 50% of lung fields (OR 4.87, p = 0.025) (Additional file 3: Table S2).

## Discussion

Our main finding was the usefulness of cfDNA levels as markers of a critical status and mortality in patients hospitalized for COVID-19, from admission and throughout the course of the disease. cfDNA also correlated with hospital stay in the survivors.

Another finding was that IL-6 was the only cytokine we studied that correlated with tissue damage markers. IL-6 levels throughout hospitalization differed completely between the critically ill and non-survivor groups on the one hand and the rest of the patients on the other. This finding could help identify patients who will progress to more severe forms and mortality, especially between days 10 and 16 from symptom onset, and those who would benefit from IL-6 blockade.

LDH, which is commonly used as a clinical marker of tissue damage, showed variations parallel to those of IL-6 and was related to severity, although its correlation with standard severity markers was clearly lower than that of cfDNA.cfDNA is a tissue damage marker released after cell destruction and, like LDH, acts as a DAMP [12], contributing to cytokine production, apoptosis, and tissue damage via Toll-like receptor 9, and is attenuated with specific Toll-like receptor 9 inhibitors. In healthy individuals, cfDNA is present in the circulation in small amounts [13], has a short life [14], and is derived primarily from hematopoietic cells. cfDNA, including nuclear and mitochondrial-derived cfDNA, is released following apoptosis and necrosis and is secreted by cells [15]. Elevated cfDNA levels indicate disease before clinical manifestations and histopathological changes [16] The main sources of cfDNA in hospitalized COVID-19 patients are hematopoietic cells, vascular endothelium, hepatocytes, adipocytes, and kidney, heart, and lung cells [16]. Non-hematopoietic cell derivative levels are 10–1000 times higher in COVID-19 patients than in healthy individuals, with levels up to 11 times higher than in hospitalized patients with influenza or respiratory syncytial virus  [16]. We found elevated cfDNA levels in the COVID-19 patients, with a significant correlation with the standard parameters of severity  [16–18]. As in other studies, our non-survivor group also showed higher cfDNA levels in the first sample after admission [7, 16, 18, 19] and thereafter. cfDNA was the only biomarker whose concentrations remained higher in these patients during the 3 disease phases, increasing steadily (especially in the late inflammatory phase), quadrupling its initial values while remaining stable in the survivors. The sustained increase in cfDNA levels also significantly predicted disease progression [7]. cfDNA was therefore the marker best related to severity and mortality, tissue damage, and outcomes in COVID-19 and was an independent risk factor for ICU admission and mortality, as shown for severity by another study [16]. At admission, cfDNA especially identified the patients at risk for critical illness and death.

In our longitudinal study, non-critical and surviving patients did not present median IL-6 values higher than 40 pg/mL at any time during their progression. In these patients, there was an large, sustained, and significant decrease in IL-6 levels on days 10–16, which was not observed in the CI patients and non-survivors, in whom the levels remained elevated or increased further. One study associated patients with baseline IL-6 concentrations above 40 pg/mL with an increased likelihood of disease progression [20]. There is increasing evidence of the importance of achieving viral control for favorable disease progression [21, 22] and of an association between the persistence of a high viral load in respiratory samples [23, 24] and in plasma [25, 26], with mortality and other unfavorable outcomes. It has been observed that, at week 1 of progression, there were no differences in viral load between ICU-ventilated and non-ventilated patients; however, the viral load decreased at week 2 in the non-ventilated patients, while it remained high in the ventilated patients [24]. One study showed that viral clearance occurred around day 10 in 90% of non-severe cases and remained positive in the most severe patients [23]. Although the gold standard for virus viability is its culture, one of the few studies available confirmed the coinciding of viable virus with high viral loads within the first 10 days [19]. There is also evidence of an association between respiratory cell infection with the release of large amounts of IL-6, which was halted with Remdesivir [27], and of a relationship between RNAemia with increased IL-6 levels [26] and lastly of a correlation between IL-6 levels and viral load in critically ill patients [28–31]. The scarce longitudinal data on cytokines in COVID-19 patients include decrease levels of IL-6 in survivors  [29], of other proinflammatory cytokines by day 16 [30], and of IL-6 two days after convalescent plasma transfusion [32]. Viral loads correlated with elevated cytokines, declining steadily in the MI, especially after day 10 [33]. We observed decreased IL-6 levels on days 10–16, suggesting an earlier viral control in our less ill patients. In critical patients and non-survivors, persistent infection would lead to more prolonged inflammation (IL-6), neutrophil activation (G-CSF and IL-8), and tissue damage (cfDNA). The exclusion criteria included concomitant infection during progression; therefore, none of the patients included in our study showed clinical, analytical, or radiological changes attributable to nosocomial infection, and the differences in IL-6 levels could not be attributed to this circumstance.

The correct selection of patients who will benefit from treatment with IL-6 blockade [9, 34–36] and its timing decreases the probability of its low utility [9, 37–39]; however, inadequate immunosuppression can also impair virus clearance [40], increase the selection of variants of concern [41], and increase the risk of infections [42]. A decrease in anti-SARS-CoV-2 neutralizing antibody activity has been reported in critical patients treated with IL-6 and IL-1 inhibitors who recovered [43]. IL-6 blocking has been recommended in severely and critically ill hospitalized patients with elevated inflammatory markers, especially CRP > 75 mg/L [44, 45]. Studies have reported that treatment is more effective when started early [8, 29, 34, 46]. Our study suggests that increased IL-6 levels > 40 pg/mL are related to a higher mortality risk. The lack of an at least threefold decrease in IL-6 from an elevated baseline on days 10–16 could indicate persistent infection and the need to start IL-6 blockade, perhaps in association with antiviral therapy, regardless of a low severity as assessed by clinical criteria. Although several COVID-19 phenotypes have been proposed, the 3 disease phases included in the study were proposed at the beginning of the pandemic, at the time of our study. However, the importance of the disease’s time course persists, for prognostic and therapeutic purposes (Additional files 1, 14).

LDH, the other tissue damage marker, is related to intracellular infection, with the release of proteins and cytoplasmic material and DNA fragmentation. LDH is a marker of tissue damage, especially in the lungs, heart, and hematopoietic cells [9]. The correlations and parallelism of IL-6 and LDH that we observed (Additional file 11: Table S9) suggest that the decrease in LDH levels in less severely ill patients (less inflammation and tissue damage) occurs after viral control is achieved. The IL-6/LDH association appeared to better reflect the inflammatory situation at various disease stages, probably as a function of viral control, while cfDNA probably more broadly reflects tissue damage [7] and is therefore more related to severity and mortality. The association between SARS-CoV-2 viral load in plasma and immune response dysregulation with increased proinflammatory cytokines (IL-6), markers of tissue damage (LDH, GPT), and critical illness has been demonstrated [47]. However, the relationship between LDH and severity/mortality was clearly less strong than that of cfDNA.

A discussion of the relationship between comorbidities, lymphopenia, CT CD4 + -derived cytokines, macrophages, pathogen-associated molecular patterns (PAMPs), DAMPs, and the cytokine storm, corticosteroids, severity, and mortality can be found in Additional file 13: Discussion.

Our study has several strengths. There have been few studies on proinflammatory cytokines and markers of tissue damage throughout the progression of COVID-19 patients. Longitudinal studies can monitor infection response dysregulation and observe its trends [4]. Changes in these parameters reveal disease dynamics and show a course towards recovery or worsening [7]. As far as we know, ours is the first study to combine these parameters with those used in the clinical management of hospitalized COVID-19 patients, according to survival and the varying severity and across the various disease phases. The Additional file 15 “Availability of data and materials.DATANOTES.pdf” is currently available [48].

Our study also has certain limitations. The cohort was recruited from a single hospital, was small, and did not include COVID-19-related coagulation disorders. Problems in assessing cytokine levels included difficulties distinguishing a vigorous beneficial immune response from a dysregulated reaction, their short half-life, and the lack of thresholds to consider an increase as abnormal [4]. Whether the primary problem is immune hyperactivity or a failure to resolve the inflammatory response due to persistent viral replication is unclear; it is likely that both were involved. Another limitation is that we could not measure the progression of viral loads in the respiratory samples or in plasma and relating them to the study parameters.

## Conclusions

Circulating cfDNA has been shown, from symptom onset and throughout the disease, to be an excellent prognostic and progression marker. The progression of IL-6 levels, especially on days 10–16 from disease onset, also provides important prognostic information, indicating that the monitoring of both markers is highly useful for the follow-up of hospitalized patients and with potential implications for their clinical management.

## Supplementary Information

**Additional file 1.** Results.

**Additional file 2: Table S1.** Variables of patients aged > 60 years, male patients, and those with some comorbidity as compared with the other patients.

**Additional file 3: Table S2.** Multivariate model for outcomes.

**Additional file 4: Table S3.** Biomarkers and respiratory severity parameters.

**Additional file 5: Table S4.** Lymphocyte count and neutrophil/lymphocyte ratio according to severity as measured by the two scores.

**Additional file 6: Table S5.** Lymphocyte count and neutrophil/lymphocyte ratio according to mortality and the 3 disease progression phases.

**Additional file 7: Table S6.** Lymphocyte count in the longitudinal study.

**Additional file 8: Figure S1.** Graphical representation of the correlations between the markers in the 3 phases of the disease. A: viral; B: early inflammatory; C. late inflammatory.

**Additional file 9: Table S7.** Comparison of biomarkers in the 3 severity groups.

**Additional file 10: Table S8.** Interleukin-6and lactate dehydrogenasevalues during the 3 disease progression phases and comparison of IL-6 and LDH levels in each phase between the survivors and non-survivors.

**Additional file 11: Table S9.** Progression of IL-6 and LDH levels through the 3 disease phases and their correlations in the varying degrees of severity according to the CCDC and WHO scales.

**Additional file 12: Table S10.** Variables in samples obtained during days 1–9 with or without corticosteroids.

**Additional file 13.** Discussion.

**Additional file 14.** References.

**Additional file 15.** Database.

## Data Availability

The datasets supporting the conclusions of this article are available in the Zenodo repository, in https://zenodo.org/record/7099678#.Yyrg1NpByUk

## Abbreviations

LDH: Lactate dehydrogenase
cfDNA: Cell free DNA
TNF-α: Tumor necrosis factor-α
G-CSF: Granulocyte colony-stimulating factor
CRP: C-reactive protein
DAMPS: Damage-associated molecular patterns
CCDC score: Chinese Center for Disease Control and Prevention classification
WHO OS: World Health Organization Ordinal Scale
MI: Moderately ill
SI: Severely ill
CI: Critically ill
IL: Interleukin
IFN-γ: Interferon-γ
PCT: Procalcitonin
SaO_2_: Oxygen saturation
FiO_2_: Fraction of inspired oxygen
GPT: Glutamic pyruvic transaminase
ICU: Intensive care unit
ARDS: Acute respiratory distress syndrome
EDTA: Ethylenediaminetetraacetic acid
