# Supplementary material for: IL-6 and cfDNA monitoring throughout COVID-19 hospitalization are accurate markers of its outcomes
Source: Respir Res. 2023 May 5;24:125. doi: 10.1186/s12931-023-02426-1 (PMC10161166; doi:10.1186/s12931-023-02426-1)
Supplement: Supplementary file 1 — Additional file 1. Results. [file 12931_2023_2426_MOESM1_ESM.docx]

**Additional file 1. docx**

**Supplementary Results**

The male COVID-19 patients in our study had higher levels of certain markers than the female patients [CRP (p=0.037), procalcitonin (p<0.001), LDH (p=0.033), D-dimer (p<0.001), ferritin (p<0.001), IL-6 (p=0.048) and cfDNA (p<0.001)]. In the first sample after admission, D-dimer, ferritin, and cfDNA levels were increased in the men; in successive samples, the other markers also increased more in the men, especially in the early inflammatory phase (days 10–16). Additional Table 1 shows the differences between the different markers in patients with and without comorbidities.

Advanced age, male sex, and obesity were associated with greater inflammatory responses and tissue damage. In the multivariate study, obesity was a risk factor for a critical condition (odds ratio [OR] 9.25, p=0.019) and ICU admission (OR 46.21, p=0.011) (Additional Table 2). Lymphopenia was always related to severity/mortality and correlated with tissue damage markers. Lymphocyte count lowered with mortality and as severity progressed (Additional Table 3). Additional Table 4 shows lymphocyte count and N/L ratio according to mortality and the three disease progression phases.

Age correlated with leukopenia, D-dimer, ferritin, and IL-8 levels in the overall samples, while the strongest and most sustained correlation throughout the progression was between age and lymphopenia. Patients older than 60 years had increased neutrophil counts (p=0.020), CRP (p=0.001), procalcitonin (p=0.002), D-dimer (p<0.001), IL-8 (p=0.001), IL-6 (p<0.001), and cfDNA levels (p=0.017), and lymphopenia (p<0.001). Patients with hypertension showed significantly increased procalcitonin, D-dimer, ferritin, IL-8, and IL-6 levels. The patients with diabetes, liver disease, and dyslipemia showed no differences in any marker.

The patients with obesity had higher leukocyte counts (p=0.003), neutrophil counts (p=0.014), and TNF-α (p=0.004) and IL-6 levels (p=0.009). The patients with heart disease showed only increased TNF-α levels (p=0.002), while the patients with chronic respiratory disease had higher neutrophil counts (p=0.028), and levels of procalcitonin (p=0.004), IL-1β (p=0.002), IFN-γ (p=0.035), and neutrophil activation markers IL-8 (p=0.044) and G-CSF (p=0.001). Renal failure in the COVID-19 patients was associated with lower platelet values (p=0.002), while the patients with dementia had strikingly lower lymphocyte counts (p=0.001). Overall, the patients with some comorbidity showed significantly higher leucocyte (p=0.045) and neutrophil counts (p=0.003), lymphopenia (p<0.001), neutrophil/lymphocyte ratio (p≤0.001), and CRP (p=0.004), procalcitonin (p=0.016), LDH (p=0.003), D-dimer (p=0.002), ferritin (p=0.004), TNF-α (p=0.018), IL-8 (p<0.001), IL-6 (p<0.001), and cfDNA levels (p=0.013) (Additional Table 1). In the first sample from the patients with some comorbidity, all differences were maintained, except TNF-α.

CRP correlated with the degree of severity and mortality, with significantly higher values in the most severely ill patients according to the CCDC score (p<0.001) and WHO OS (p= 0.020), and in the non-survivors (p<0.001), from the first sample at admission (p=0.016) and during the entire hospital stay. In the CI and non-survivors, there was a sharp increase in CRP from day 17 onwards, while there was a sustained decrease from their initial high values in the SI (p=0.001, WHO OS) and MIG (p=0.004, CCDC score) survivors through both inflammatory phases. CRP correlated significantly with IL-6 in the first sample after admission (r=0.437, p=0.000), in the viral phase (r=0.414, p=0.000), and in the early inflammatory phase (0.628, p=0.000) but not in the late phase. CRP and IL-6 were also correlated in the MI and SI (r=0.29, p=0.001) but not in the CI group.

The SI and especially the CI groups had higher procalcitonin levels in both scores (p>0.001, CDC and p=0.009, WHO OS) than the MI. The non-survivors also had significantly higher procalcitonin levels (p<0.001), and the differences in terms of mortality became more evident as the disease progressed (p=0.046). Ferritin levels were significantly higher in the SI and CI than in the MI group in both scales (p<0.001 for CDC and p=0.001 for WHO OS), as well as in the non-survivors (p=0.002). Although the overall rate of all patients, ferritin increased slightly in the 10–16-day period (p=0.048), it remained stable in the MI group, while in the SI, the rate fell from day 17 onwards (p=0.009). Conversely, ferritin levels continued to increase in the CI throughout the disease progression, although with no statistically significant differences. Additional Table 3 shows the relationship between certain analytical parameters and SaO_2_, SaO_2_/FiO_2_ and the extent of pulmonary infiltrates.

The lymphocyte counts were progressively lower and the neutrophil/lymphocyte ratios (N/L ratio) were progressively higher as the severity increased (p<0.001) (Additional Table 4). The survivors had twice the lymphocyte count of the non-survivors (p<0.001), from the first sample taken at hospital admission to the sample taken more than 17 days after symptom onset (Additional Table 5). The N/L ratio was 4-fold higher for the non-survivors than for the survivors (p<0.001) (Additional Table 5). The lymphocyte count was 2 times higher in the survivors than in the non-survivors in the viral and early inflammatory phases and 3 times higher than in those who died in the late inflammatory phase (Additional Table 5). Lymphocyte counts remained stable throughout the hospital stay when the samples from all patients were assessed. However, differences were observed between the 3 severity levels in the 72 patients with several samples. The MI group showed normal and stable lymphocyte counts across the 3 disease progression stages. The SI initially had lymphopenia (median 800/mm^3^), which normalised in the late inflammatory phase (>17 days) (p=0.003, for severe group of the WHO OS), whereas the CI and non-survivors started with very low lymphocyte counts (median 500/mm^3^), which did not increase throughout the hospital stay (Additional Table 6). Lymphopenia was significantly correlated with other markers of inflammation and severity (IL-6) and tissue damage, such as the N/L ratio, IL-6, LDH, and cfDNA.

We found close correlations between TNF-α, IL-1β, IL-8, and IFN-γ levels in all severity grades and during the 3 phases, as well as a high correlation between TNF-α and both IL-17A and IFN-γ in all degrees of severity and in the viral and inflammatory phases (r=0.746, p=0.000 for IFN-γ and r=0.700, p=0.000 for IL-17A) (Additional Figure 1). In the patients who became critical or died, there was no sustained increase in IFN-γ levels, which we observed in the less severely ill patients (p<0.001).
