# Supplementary material for: IL-6 and cfDNA monitoring throughout COVID-19 hospitalization are accurate markers of its outcomes
Source: Respir Res. 2023 May 5;24:125. doi: 10.1186/s12931-023-02426-1 (PMC10161166; doi:10.1186/s12931-023-02426-1)
Supplement: Supplementary file 2 — Additional file 2: Table S1. Variables of patients aged > 60 years, male patients, and those with some comorbidity as compared with the other patients. [file 12931_2023_2426_MOESM2_ESM.docx]

Additional file 2.docx

Supplementary Table 1

Supplementary Table 1: Variables of patients aged >60 years, male patients, and those with some comorbidity as compared with the other patients (total samples). Abbreviations: N/L ratio: neutrophils/lymphocytes ratio; CRP: C-reactive protein; PCT: procalcitonin; LDH: lactate dehydrogenase; TNF-α: Tumor necrosis factor-α; IL-8: Interleukin-8; IL-1β: interleukin-1β; IFN-γ: interferon- γ: IL-17A: intereleukin-17A; G-CSF: Granulocyte colony-stimulating factor; IL-6: Interleukin-6; cfDNA: cell free DNA; SaO2/ FiO2: oxygen saturation/fraction of inspired oxygen; SaO2: oxygen saturation.

|  | **No comorbidities**  **N=31** | **≥ 1 comorbidities**  **N=170** | **p-value** |
| --- | --- | --- | --- |
| **Leukocytes/mm3** | 6700 [5100;9050] | 7850 [6100;10175] | 0.042 |
| **Neutrophils/mm3** | 4400 [2150;6400] | 5850 [4025;8200] | 0.002 |
| **Lymphocytes/mm3** | 2100 [1200;2400] | 1000 [600;1400] | <0.001 |
| **N/L ratio** | 1.90 [1.22;4.44] | 5.91 [2.93;11.57] | <0.001 |
| **Platelet/mm3** | 248000 [227000;325500] | 235000 [180000;316750] | 0.218 |
| **CRP (mg/L)** | 7.65 [2.52;61.80] | 34.00 [12.10;95.10] | 0.003 |
| **PCT (ng/mL)** | 0.04 [0.04;0.11] | 0.08 [0.05;0.14] | 0.015 |
| **LDH (U/L)** | 206.00 [171.00;281.00] | 277.50 [214.50;368.75] | 0.003 |
| **D-Dimer (µg/L)** | 597.00 [328.00;865.00] | 871.00 [471.50;1665.00] | 0.002 |
| **Ferritin (ng/mL)** | 318.90 [109.10;515.10] | 497.10 [280.52;837.30] | 0.004 |
| **TNF-α (pg/mL)** | 36.45 [33.58;41.83] | 40.80 [35.40;48.63] | 0.018 |
| **IL-8 (pg/mL)** | 41.42 [33.42;55.43] | 56.24 [42.39;94.72] | <0.001 |
| **IL-1β (pg/mL)** | 53.87 [45.68;65.74] | 56.31 [47.80;67.21] | 0.559 |
| **IFN-ʏ (pg/mL)** | 138.34 [125.53;175.10] | 146.99 [124.01;177.03] | 0.726 |
| **IL-17A (pg/mL)** | 18.94 [11.77;23.63] | 19.40 [14.78;24.16] | 0.158 |
| **P-Selectin (ng/mL)** | 56.99 [41.56;67.96] | 55.19 [42.94;74.07] | 0.629 |
| **G-CSF (pg/mL)** | 133.90 [120.63;153.66] | 144.93 [124.90;161.48] | 0.178 |
| **IL-6 (pg/mL)** | 2.70 [1.50;5.96] | 26.30 [5.77;58.60] | <0.001 |
| **cfDNA (ng/mL)** | 6.14 [2.81;9.47] | 8.03 [4.63;15.02] | 0.013 |
| **SaO2/FiO2** | 462.00 [457.00;471.00] | 428.00 [333.75;452.00] | <0.001 |
| **SaO2** | 97.00 [93.00;99.00] | 92.00 [87.00;95.00] | <0.001 |
