# Supplementary material for: IL-6 and cfDNA monitoring throughout COVID-19 hospitalization are accurate markers of its outcomes
Source: Respir Res. 2023 May 5;24:125. doi: 10.1186/s12931-023-02426-1 (PMC10161166; doi:10.1186/s12931-023-02426-1)
Supplement: Supplementary file 3 — Additional file 3: Table S2. Multivariate model for outcomes. [file 12931_2023_2426_MOESM3_ESM.docx]

Additional file 3.docx

Supplementary Table 2

Supplementary Table 2: Multivariate model for outcomes. Abbreviations: Interleukin-6; cfDNA: cell free DNA;

| **CDC score** | **Moderate and Severe** | **Critical** | **OR (univariate)** | **OR (multivariate)** |
| --- | --- | --- | --- | --- |
| Age>60  n (%) | 37 (61.7) | 23 (38.3) | 4.51 (1.53-16.66, p=0.012) | 2.70 (0.63-14.33, p=0.201) |
| Male sex  n (%) | 25 (59.5) | 17 (40.5) | 2.79 (1.12-7.24, p=0.030) | 2.51 (0.60-12.03, p=0.216) |
| Hypertension  n (%) | 29 (64.4) | 16 (35.6) | 1.86 (0.75-4.70, p=0.182) | - |
| **Obesity**  **n (%)** | **5 (38.5)** | **8 (61.5)** | **5.14 (1.53-18.82, p=0.009)** | **9.25 (1.54-67.46, p=0.019)** |
| Diabetes  n (%) | 21 (75.0) | 7 (25.0) | 0.75 (0.26-1.99, p=0.575) | - |
| IL-6  Mean (SD) | 45.5 (118.1) | 170.7 (195.5) | 1.01 (1.00-1.01, p=0.009) | 1.00 (1.00-1.01, p=0.195) |
| cfDNA  Mean (SD) | 8.0 (6.1) | 17.4 (14.1) | 1.12 (1.05-1.20, p=0.001) | 1.07 (1.01-1.16, p=0.055) |
| Corticosteroids therapy n (%) | 28 (59.6) | 19 (40.4) | 3.22 (1.27-8.81, p=0.0171) | 1.76 (0.49-6.64, p=0.385) |
| **Death** | **No** | **Yes** | **OR (univariate)** | **OR (multivariate)** |
| Age>60  n (%) | 43 (71.7) | 17 (28.3) | 6.13 (1.60-40.44, p=0.021) | 3.17 (0.58-27.61, p=0.223) |
| Male sex  n (%) | 31 (73.8) | 11 (26.2) | 1.91 (0.69-5.46, p=0.215) | 1.10 (0.26-4.62, p=0.891) |
| Hypertension  n (%) | 31 (68.9) | 14 (31.1) | 3.88 (1.33-13.06, p=0.018) | 2.87 (0.68-15.24, p=0.175) |
| Obesity  n (%) | 8 (61.5) | 5 (38.5) | 2.95 (0.79-10.27, p=0.092) | 2.25 (0.45-11.24, p=0.314) |
| Diabetes  n (%) | 25 (89.3) | 3 (10.7) | 0.37 (0.08-1.23, p=0.138) | - |
| IL-6  Mean (SD) | 72.2 (159.0) | 113.2 (119.3) | 1.00 (1.00-1.00, p=0.344) | - |
| **cfDNA**  **Mean (SD)** | **8.8 (8.0)** | **17.5 (13.1)** | **1.09 (1.03-1.16, p=0.006)** | **1.08 (1.02-1.15, p=0.014)** |
| Corticosteroids therapy n (%) | 35 (74.5) | 12 (25.5) | 1.91 (0.69-5.64, p=0.222) | - |
| **ICU admittance** | **No** | **Yes** | **OR (univariate)** | **OR (multivariate)** |
| Age>60  n (%) | 51 (85.0) | 9 (15.0) | 1.76 (0.48-8.41, p=0.421) | 0.69 (0.05-10.85, p=0.780) |
| Male sex  n (%) | 34 (85.0) | 8 (19.0) | 2.76 (0.80-11.06, p=0.119) | 1.84 (0.08-63.05, p=0.695) |
| Hypertension  n (%) | 40 (88.9) | 5 (11.1) | 0.73 (0.20-2.48, p=0.619) | - |
| **Obesity**  **n (%)** | **7 (53.8)** | **6 (46.2)** | **10.57 (2.69-43.67, p=0.001)** | **46.21 (3.76-2138.71, p=0.011)** |
| Diabetes  n (%) | 25 (89.3) | 3 (10.7) | 0.75 (0.16-2.75, p=0.680) | - |
| IL-6  Mean (SD) | 51.2 (112.2) | 260.1 (240.1) | 1.01 (1.00-1.01, p=0.002) | 1.00 (1.00-1.01, p=0.102) |
| cfDNA  Mean (SD) | **8.7 (6.5)** | **23.7 (17.4)** | **1.14 (1.06-1.25, p=0.001)** | **1.22 (1.07-1.53, p=0.025)** |
| Corticosteroids therapy n (%) | 36 (76.6) | 11 (23.4) | 13.75 (2.50-257.24, p=0.014) | 19.62 (1.07-2584.67, p=0.114) |
