# Supplementary material for: IL-6 and cfDNA monitoring throughout COVID-19 hospitalization are accurate markers of its outcomes
Source: Respir Res. 2023 May 5;24:125. doi: 10.1186/s12931-023-02426-1 (PMC10161166; doi:10.1186/s12931-023-02426-1)
Supplement: Supplementary file 4 — Additional file 4: Table S3. Biomarkers and respiratory severity parameters. [file 12931_2023_2426_MOESM4_ESM.docx]

Additional file 4.docx

Supplementary Table 3

Supplementary Table 3: Biomarkers and respiratory severity parameters. Abbreviations: LDH: lactate dehydrogenase; CRP: C-reactive protein; PCT: procalcitonin; IL-6: Interleukin-6; cfDNA: cell free DNA; SaO2/ FiO2: oxygen saturation/fraction of inspired oxygen; SaO2: oxygen saturation.

| **Analytical**    **Clinical** | **LDH**  **(U/L)** | **CRP**  **(mg/L)** | **PCT**  **(ng/mL)** | **Ferritin**  **(ng/mL)** | **Lymphocyte**  **(mm^3^)** | **IL-6**  **(pg/mL)** | **Cf-DNA**  **(ng/mL)** |
| --- | --- | --- | --- | --- | --- | --- | --- |
| **Oxygen saturation(SaO_2_)** |  |  |  |  |  |  |  |
| > 93%  N = 96 | 227  (188-306) | 20,3  (7,6-66) | 0,06  (0,04-0,11) | 344  (160,9-555,5) | 1200  (900-1825) | 7,98  (3,49-33,58) | 6.20  (3.61-10.87) |
| ≤93%  N= 76 | 325  (235-397) | 39,4  (11,9-117,3) | 0,09  (0,05-0,18) | 624,19  (390,85-1165) | 900  (675-1425) | 33,3  (5,83-83,83) | 9.75  (6.48-17.79) |
|  | p<0,001 | p=0,011 | p=0,002 | p<0,001 | p=0,003 | p<0,001 | p=0,001 |
| **SaO_2_/FiO_2_** |  |  |  |  |  |  |  |
| >315  N=117 | 233  (189,75-315,25) | 21,75  (7,75-67,2) | 0,06  (0,04-0,11) | 380,2  (197,7-602) | 1200  (900-1800) | 10,53  (3,49-33,52) | 6.77  (3.52-10.18) |
| ≤315  N=60 | 316  (238-394) | 78,4  (25,24-151,8) | 0,09  (0,05-0,19) | 626,4  (445-1002,75) | 700  (400-925) | 33,24  (5,01-57,59) | 10.50  (6.11-20.75) |
|  | p<0,001 | p<0,001 | p=0,045 | p<0,001 | p<0,001 | p=0,011 | p<0,001 |
| **Occupation of Infiltrates in**  **chest X-ray** |  |  |  |  |  |  |  |
| ≤50%  N=160 | 247  (194-326,5) | 27,25  (9,55-81,85) | 0,07  (0,04-0,11) | 430,15  (296,95-646,5) | 1100  (700-1725) | 23,04  (5,99-44,87) | 7.10  (4.25-11.40) |
| >50%  N=40 | 358  (286,5-440) | 78,4  (9,6-210) | 0,11  (0,05-0,29) | 980  (524,75-1351,45) | 800  (400-1200) | 60,86  (5,49-171,95) | 14.14  (7.46-19.49) |
|  | p<0,001 | p=0,037 | p=0,14 | p<0,001 | p=0,002 | p=0,046 | p<0,001 |
