# Supplementary material for: IL-6 and cfDNA monitoring throughout COVID-19 hospitalization are accurate markers of its outcomes
Source: Respir Res. 2023 May 5;24:125. doi: 10.1186/s12931-023-02426-1 (PMC10161166; doi:10.1186/s12931-023-02426-1)
Supplement: Supplementary file 5 — Additional file 5: Table S4. Lymphocyte count and neutrophil/lymphocyte ratio according to severity as measured by the two scores. [file 12931_2023_2426_MOESM5_ESM.docx]

Additional file 5.docx

Supplementary Table 4

Supplementary Table 4: Lymphocyte count and neutrophil/lymphocyte ratio according to severity as measured by the two scores. Abbreviations: N/L ratio: neutrophils/lymphocytes ratio.

| **CDC score** | | | | | | | | |
| --- | --- | --- | --- | --- | --- | --- | --- | --- |
| **Severity** | **Moderate** | **Severe** | | **Critical** | **p-value** | |  |  |
| **Lymphocytes /mm3** | 1200 [900;2000] | 1000 [700;1400] | | 700 [400;1000] | <0.001 | |  |  |
| **N/L Ratio** | 3.64 [1.76;6.40] | 5.06 [2.88;9.57] | | 15.31 [6.51;28.69] | <0.001 | |  |  |
| **WHO OS** | | | | | | | | |
| **Severity** | **Moderate** | | **Severe/Critical** | | | **p-value** | |  |
| **Lymphocytes /mm3** | 1200 [950;1800] | | 800 [500;1375] | | | <0.001 | |  |
| **N/L Ratio** | 3.53 [1.95;6.58] | | 8.00 [4.25;18.00] | | | <0.001 | |  |
