# Supplementary material for: IL-6 and cfDNA monitoring throughout COVID-19 hospitalization are accurate markers of its outcomes
Source: Respir Res. 2023 May 5;24:125. doi: 10.1186/s12931-023-02426-1 (PMC10161166; doi:10.1186/s12931-023-02426-1)
Supplement: Supplementary file 6 — Additional file 6: Table S5. Lymphocyte count and neutrophil/lymphocyte ratio according to mortality and the 3 disease progression phases. [file 12931_2023_2426_MOESM6_ESM.docx]

Additional file 6.docx

Supplementary Table 5

Supplementary Table 5: Lymphocyte count and neutrophil/lymphocyte ratio according to mortality and the 3 disease progression phases. Abbreviations: N/L ratio: neutrophils/lymphocytes ratio.

|  | **Survivors** | **Non-survivors** | **p-value** |
| --- | --- | --- | --- |
| **Lymphocytes /mm3** | 1200 [800;1800] | 600 [400;800] | <0.001 |
| **N/L Ratio** | 4.29 [2.23;7.49] | 15.80 [7.85;29.98] | <0.001 |
| **Longitudinal Study** | **Lymphocyte count - Survivors** | **Lymphocyte count – Non-survivors** | **p-value** |
| **1-9 days** | 1200 [800;1600] | 600 [450;800] | <0.001 |
| **10-16 days** | 1200 [775;1800] | 700 [400;800] | 0.002 |
| **>17 days** | 1300 [800;2300] | 500.00 [375;1000] | 0.001 |
