# Supplementary material for: IL-6 and cfDNA monitoring throughout COVID-19 hospitalization are accurate markers of its outcomes
Source: Respir Res. 2023 May 5;24:125. doi: 10.1186/s12931-023-02426-1 (PMC10161166; doi:10.1186/s12931-023-02426-1)
Supplement: Supplementary file 7 — Additional file 7: Table S6. Lymphocyte count in the longitudinal study. [file 12931_2023_2426_MOESM7_ESM.docx]

Additional file 7.docx

Supplementary Table 6

Supplementary Table 6: Lymphocyte count in the longitudinal study. Abbreviations: CCDC score: Chinese Center for Disease Control and Prevention classification; WHO OS: World Health Organisation Ordinal Scale

| **CCDC score** | - 1. **days** | - 1. **days** | **>17 days** | **p-value** |
| --- | --- | --- | --- | --- |
| **Moderate** | 1200 [950;1800] | 1200 [825;1775] | 1400 [950;2300] | 0.502 |
| **Severe** | 900 [600;1300] | 1300 [650;1900] | 1400 [1075;2175] | 0.125 |
| **Critical** | 500 [400;800] | 700 [400;800] | 600 [400;1300] | 0.760 |
| **WHO OS** | **1-9 days** | **10-16 days** | **>17 days** | **p-value** |
| **Moderate** | 1200 [1000;1650] | 1300 [775;1775] | 1100 [800;1400] | 0.755 |
| **Severe** | 800 [600;1300] | 900 [700;1550] | 2250 [1525;2825] | 0.003 |
| **Critical** | 500 [350;725] | 700 [400;800] | 600 [400;1300] | 0.729 |
| **Death** | **1-9 days** | **10-16 days** | **>17 days** | **p-value** |
| **No** | 1200 [800;1575] | 1150 [700;1775] | 1400 [800;2300] | 0.328 |
| **Yes** | 565 [400;800] | 700 [400;800] | 400 [350;1100] | 0.981 |
