# Supplementary material for: IL-6 and cfDNA monitoring throughout COVID-19 hospitalization are accurate markers of its outcomes
Source: Respir Res. 2023 May 5;24:125. doi: 10.1186/s12931-023-02426-1 (PMC10161166; doi:10.1186/s12931-023-02426-1)

Additional file 8. docx

Supplementary Figure 1

Supplementary Figure 1: Graphical representation of the correlations between the markers in the 3 phases of the disease. A: viral; B: early inflammatory; C. late inflammatory

A


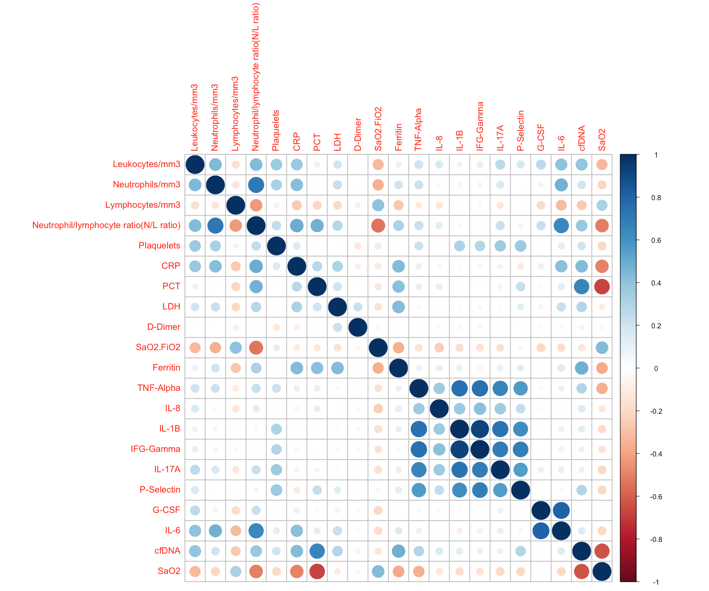


B


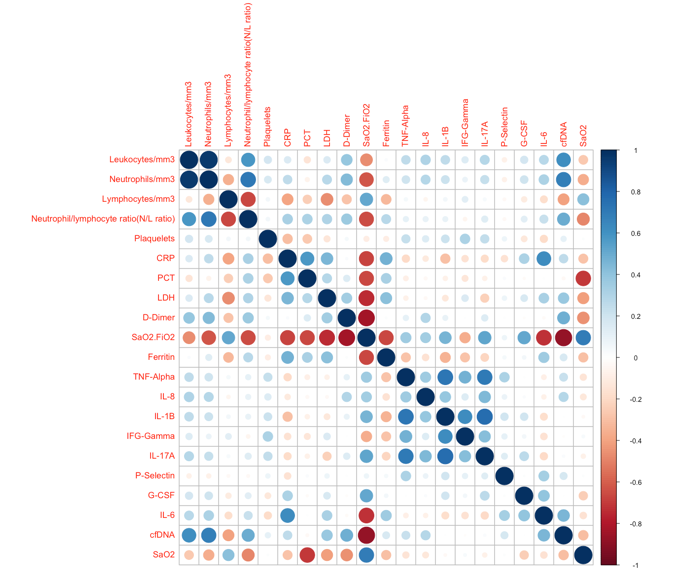


C


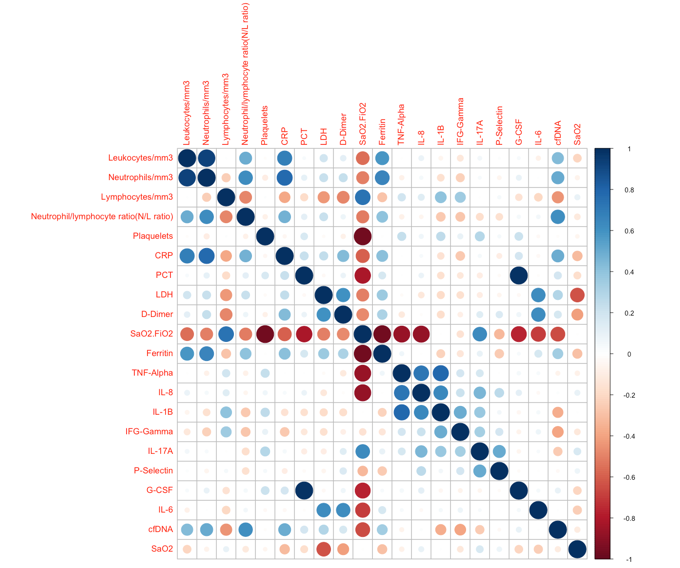

Supplement: Supplementary file 8 — Additional file 8: Figure S1. Graphical representation of the correlations between the markers in the 3 phases of the disease. A: viral; B: early inflammatory; C. late inflammatory. [file 12931_2023_2426_MOESM8_ESM.docx]
