# Supplementary material for: IL-6 and cfDNA monitoring throughout COVID-19 hospitalization are accurate markers of its outcomes
Source: Respir Res. 2023 May 5;24:125. doi: 10.1186/s12931-023-02426-1 (PMC10161166; doi:10.1186/s12931-023-02426-1)
Supplement: Supplementary file 9 — Additional file 9: Table S7. Comparison of biomarkers in the 3 severity groups. [file 12931_2023_2426_MOESM9_ESM.docx]

Additional file 9.docx

Supplementary Table 7

Supplementary Table 7: Comparison of biomarkers in the 3 severity groups (Chinese Center for Diseases Control score). Abbreviations: N/L ratio: neutrophils/lymphocytes ratio; CRP: C-reactive protein; PCT: procalcitonin; LDH: lactate dehydrogenase; TNF-α: Tumor necrosis factor-α; IL-8: Interleukin-8; IL-1β: interleukin-1β; IFN-γ: interferon- γ: IL-17A: intereleukin-17A; G-CSF: Granulocyte colony-stimulating factor; IL-6: Interleukin-6; cfDNA: cell free DNA; SaO2/ FiO2: oxygen saturation/fraction of inspired oxygen; SaO2: oxygen saturation.

| **CDC scale** | **1**  **N=105** | **2**  **N=38** | **3**  **N=58** | **p-value** | **p-value 1 vs 2** | **p-value 1 vs 3** | **p-value 2 vs 3** |
| --- | --- | --- | --- | --- | --- | --- | --- |
| **Leukocytes/mm3** | 7100 [5400;8700] | 7200 [6210;8875] | 9250 [7075;13075] | <0.001 | 0.438 | <0.001 | 0.009 |
| **Neutrophils/mm3** | 4600 [3500;6400] | 5600 [4175;6850] | 8250 [5400;12100] | <0.001 | 0.087 | <0.001 | 0.001 |
| **Lymphocytes/mm3** | 1200 [900;2000] | 1150 [700;1550] | 700 [400;900] | <0.001 | 0.068 | <0.001 | 0.001 |
| **N/L ratio** | 3.64 [1.74;6.40] | 4.80 [2.76;8.73] | 15.12 [6.56;27.25] | <0.001 | 0.015 | <0.001 | <0.001 |
| **Platelet/mm3** | 249000 [187000;329000] | 224000 [180500;258000] | 229000 [173000;328500] | 0.159 | 0.147 | 0.553 | 0.377 |
| **CRP (mg/L)** | 19.95 [7.42;60.60] | 25.80 [9.40;79.40] | 100.05 [33.80;180.60] | <0.001 | 0.329 | <0.001 | 0.001 |
| **PCT (ng/mL)** | 0.05 [0.04;0.10] | 0.09 [0.06;0.11] | 0.13 [0.07;0.29] | <0.001 | 0.023 | <0.001 | 0.051 |
| **LDH (U/L)** | 226.00 [189.00;306.00] | 274.00 [224.00;345.00] | 355.00 [255.00;421.00] | <0.001 | 0.048 | <0.001 | 0.019 |
| **D-Dimer (µg/L)** | 603.50 [391.50;985.00] | 908.00 [338.00;1626.00] | 1512.00 [698.00;2392.00] | <0.001 | 0.272 | <0.001 | 0.038 |
| **Ferritin (ng/mL)** | 340.85 [136.43;510.55] | 624.15 [468.22;1126.50] | 786.80 [445.00;1368.80] | <0.001 | <0.001 | <0.001 | 0.516 |
| **TNF-α (pg/mL)** | 39.60 [34.83;45.85] | 39.60 [34.83;50.47] | 40.85 [36.26;48.84] | 0.260 | 0.658 | 0.295 | 0.704 |
| **IL-8 (pg/mL)** | 49.89 [35.59;80.94] | 55.84 [46.72;102.29] | 68.60 [47.40;94.41] | 0.047 | 0.156 | 0.072 | 0.660 |
| **IL-1β (pg/mL)** | 56.32 [47.80;65.74] | 54.04 [46.85;69.95] | 56.32 [50.89;66.79] | 0.897 | 0.881 | 0.881 | 0.881 |
| **IFN-ʏ (pg/mL)** | 149.47 [123.72;178.02] | 145.84 [119.97;182.83] | 142.20 [128.56;163.30] | 0.695 | 0.913 | 0.913 | 0.913 |
| **IL-17A (pg/mL)** | 19.40 [14.73;23.90] | 19.40 [14.95;24.16] | 19.40 [14.95;24.77] | 0.514 | 0.653 | 0.653 | 0.916 |
| **P-Selectin (ng/mL)** | 56.13 [40.26;71.35] | 56.13 [44.80;72.55] | 53.22 [43.12;84.08] | 0.918 | 0.874 | 0.874 | 0.874 |
| **G-CSF (pg/mL)** | 142.01 [122.64;156.55] | 136.15 [120.58;153.66] | 152.80 [131.66;177.42] | 0.030 | 0.631 | 0.045 | 0.045 |
| **IL-6 (pg/mL)** | 9.00 [2.76;33.84] | 10.39 [4.65;31.69] | 60.17 [20.89;178.40] | <0.001 | 0.612 | <0.001 | <0.001 |
| **cfDNA (ng/mL)** | 6.21 [3.46;10.44] | 7.45 [4.99;14.90] | 14.11 [7.13;22.67] | <0.001 | 0.088 | <0.001 | 0.012 |
| **SaO2/FiO2** | 96.00 [93.00;98.00] | 90.00 [87.00;93.00] | 85.00 [80.00;88.00] | <0.001 | <0.001 | <0.001 | <0.001 |
