# Supplementary material for: IL-6 and cfDNA monitoring throughout COVID-19 hospitalization are accurate markers of its outcomes
Source: Respir Res. 2023 May 5;24:125. doi: 10.1186/s12931-023-02426-1 (PMC10161166; doi:10.1186/s12931-023-02426-1)
Supplement: Supplementary file 10 — Additional file 10: Table S8. Interleukin-6and lactate dehydrogenasevalues during the 3 disease progression phases and comparison of IL-6 and LDH levels in each phase between the survivors and non-survivors. [file 12931_2023_2426_MOESM10_ESM.docx]

Additional file 10.docx

Supplementary Table 8

Supplementary Table 8: Interleukin-6 (IL-6) and lactate dehydrogenase (LDH) values during the 3 disease progression phases and comparison of IL-6 and LDH levels in each phase between the survivors and non-survivors.

P-value1: p-values of the comparison of the progression of the biomarker levels in the various phases (survivors only).

P-value2: p-values of the comparison of the progression of the biomarker’s levels in the various phases (non-survivors only).

| **Days of symptom onset** | | | | | | | | | | | |
| --- | --- | --- | --- | --- | --- | --- | --- | --- | --- | --- | --- |
|  | **1-9 days**  **n=97** | | | **10-16 days**  **n=59** | | | **≥17 days**  **n=45** | | **p-value** |  |  |
| **IL6 (pg/mL)** | 29.11 [6.26;58.96] | | | 8.92 [1.93;27.26] | | | 10.06 [3.20;50.89] | | 0.011 |  |  |
| **LDH (U/L)** | 294.50 [223.25;385.25] | | | 274.00 [201.00;345.00] | | | 232.00 [173.00;327.25] | | 0.127 |  |  |
|  | **Survivors**  **n=78** | **Non-survivors**  **n=19** | **p-value** | **Survivors**  **n=48** | **Non-survivors**  **n=11** | **p-value** | **Survivors**  **n=32** | **Non-survivors**  **n=13** | **p-value** | **p-value1** | **p-value2** |
| **IL6 (pg/mL)** | 27.27 [5.78;51.08] | 57.27 [31.73;98.75] | 0.039 | 5.28 [1.50;23.10] | 49.12 [26.78;214.49] | 0.001 | 8.23 [2.91;37.72] | 87.09 [5.52;199.48] | 0.114 | 0.002 | 0.801 |
| **LDH (U/L)** | 289.00 [223.00;370.00] | 323.00 [243.00;417.00] | 0.347 | 267.00 [196.25;324.00] | 358.00 [228.00;417.00] | 0.185 | 202.50 [170.75;294.75] | 378.00 [319.00;471.25] | 0.001 | 0.006 | 0.594 |
