# Supplementary material for: IL-6 and cfDNA monitoring throughout COVID-19 hospitalization are accurate markers of its outcomes
Source: Respir Res. 2023 May 5;24:125. doi: 10.1186/s12931-023-02426-1 (PMC10161166; doi:10.1186/s12931-023-02426-1)
Supplement: Supplementary file 11 — Additional file 11: Table S9. Progression of IL-6 and LDH levels through the 3 disease phases and their correlations in the varying degrees of severity according to the CCDC and WHO scales. [file 12931_2023_2426_MOESM11_ESM.docx]

Additional File 11.docx

Supplementary Table 9

Supplementary Table 9: Evolution of IL-6 and LDH levels through the 3 phases of the disease and their correlations in the different degrees of severity according to the CCDC and WHO scales.

| **CCDC scale** | **Viral phase (1)** | **Early inflammatory (2)** | **Early inflammatory (3)** | **p-value** | **p-value 1 vs 2** | **p-value 1 vs 3** | **p-value 2 vs 3** |
| --- | --- | --- | --- | --- | --- | --- | --- |
| **MODERATE** | | | | | | | |
| **IL-6 (pg/mL)** | 24.65 [6.38;41.95] | 4.19 [1.50;17.53] | 3.69 [1.50;22.83] | 0.001 | 0.004 | 0.014 | 1.000 |
| **LDH (U/L)** | 274.00 [217.00;365.25] | 225.00 [186.25;306.00] | 199.00 [168.50;220.50] | 0.003 | 0.088 | 0.004 | 0.088 |
| **SEVERE** | | | | | | | |
| **IL-6 (pg/mL)** | 18.16 [5.28;51.92] | 8.05 [2.59;19.46] | 5.91 [3.37;10.53] | 0.169 | 0.189 | 0.189 | 0.877 |
| **LDH (U/L)** | 330.00 [272.00;398.00] | 298.00 [259.25;327.00] | 175.00 [172.25;246.75] | 0.020 | 0.305 | 0.024 | 0.067 |
| **CRITICAL** | | | | | | | |
| **IL-6 (pg/mL)** | 58.38 [32.08;125.26] | 49.12 [22.18;225.32] | 152.80 [12.88;447.90] | 0.673 | 0.909 | 0.909 | 0.909 |
| **LDH (U/L)** | 323.00 [240.50;415.00] | 374.00 [283.50;430.00] | 355.00 [325.00;461.00] | 0.498 | 0.664 | 0.664 | 0.664 |
|  | | | | | | | |
| **CCDC scale** | **Moderate** | **Severe** | **Critical** |  |  |  |  |
| **IL6-LDH** | r= 0.224; p=0.031 | r= 0.315; p<0.001 | r= 0.467; p=0.002 |  |  |  |  |
| **WHO OS** | **3-4 score** | **5-6 score** | **7-8 score** |  |  |  |  |
| **IL6-LDH** | r= 0.274; p=0.009 | r= 0.417; p<0.001 | r= 0.472; p=0.004 |  |  |  |  |
