# Supplementary material for: IL-6 and cfDNA monitoring throughout COVID-19 hospitalization are accurate markers of its outcomes
Source: Respir Res. 2023 May 5;24:125. doi: 10.1186/s12931-023-02426-1 (PMC10161166; doi:10.1186/s12931-023-02426-1)
Supplement: Supplementary file 12 — Additional file 12: Table S10. Variables in samples obtained during days 1–9 with or without corticosteroids. [file 12931_2023_2426_MOESM12_ESM.docx]

Additional file 12.docx

Supplementary Table 10

Supplementary Table 10: Variables in samples obtained during days 1-9 with or without corticosteroids. Abbreviations: N/L ratio: neutrophils/lymphocytes ratio; CRP: C-reactive protein; PCT: procalcitonin; LDH: lactate dehydrogenase; TNF-α: Tumor necrosis factor-α; IL-8: Interleukin-8; IL-1β: interleukin-1β; IFN-γ: interferon- γ: IL-17A: intereleukin-17A; G-CSF: Granulocyte colony-stimulating factor; IL-6: Interleukin-6; cfDNA: cell free DNA; SaO2/ FiO2: oxygen saturation/fraction of inspired oxygen; SaO2: oxygen saturation.

| **Corticosteroids therapy** | **No**  **N=69** | **Yes**  **N=28** | **p-value** |
| --- | --- | --- | --- |
| **Leukocytes/mm3** | 7000.00 [5300.00;8900.00] | 6520.00 [4475.00;10075.00] | 0.610 |
| **Neutrophils/mm3** | 4700.00 [3400.00;6800.00] | 4900.00 [3350.00;9225.00] | 0.583 |
| **Lymphocytes/mm3** | **1200.00 [800.00;1700.00]** | **700.00 [500.00;1100.00]** | **0.001** |
| **N/L ratio** | **4.50 [2.08;7.62]** | **7.37 [3.41;22.87]** | **0.022** |
| **Platelet/mm3** | 222000.00 [166000.00;285000.00] | 235000.00 [177500.00;288250.00] | 0.656 |
| **CRP (mg/L)** | 50.00 [12.45;112.20] | 65.30 [20.73;93.90] | 0.610 |
| **PCT (ng/mL)** | 0.08 [0.05;0.15] | 0.08 [0.04;0.14] | 0.834 |
| **LDH (U/L)** | 294.50 [217.00;375.75] | 260.00 [225.00;390.50] | 0.843 |
| **D-Dimer (µg/L)** | 843.00 [406.50;1464.00] | 882.00 [610.00;1332.25] | 0.241 |
| **Ferritin (ng/mL)** | 412.40 [199.12;587.12] | 495.60 [293.20;622.90] | 0.288 |
| **TNF-α (pg/mL)** | **35.49 [31.53;41.98]** | **39.43 [35.68;47.49]** | **0.021** |
| **IL-8 (pg/mL)** | 49.24 [33.42;71.61] | 54.75 [40.01;68.12] | 0.367 |
| **IL-1β (pg/mL)** | 47.80 [42.74;62.48] | 53.60 [45.44;60.82] | 0.243 |
| **IFN-ʏ (pg/mL)** | 130.64 [114.01;159.48] | 134.86 [123.32;153.08] | 0.428 |
| **IL-17A (pg/mL)** | 15.67 [10.79;21.31] | 18.94 [11.84;23.90] | 0.289 |
| **P-Selectin (ng/mL)** | 45.88 [34.27;58.73] | 49.25 [35.17;72.09] | 0.472 |
| **G-CSF (pg/mL)** | 136.15 [120.38;157.76] | 148.51 [128.28;181.16] | 0.128 |
| **IL-6 (pg/mL)** | 28.70 [6.40;51.33] | 33.14 [5.28;81.00] | 0.684 |
| **cfDNA (ng/mL)** | 6122.78 [3833.83;13123.42] | 7192.02 [4875.34;13901.22] | 0.339 |
| **SaO2** | **95.00 [90.00;97.00]** | **90.00 [85.00;95.00]** | **0.025** |
| **SaO2.FiO2** | 447.00 [380.00;462.00] | 430.50 [354.50;450.75] | 0.200 |
