# Supplementary material for: IL-6 and cfDNA monitoring throughout COVID-19 hospitalization are accurate markers of its outcomes
Source: Respir Res. 2023 May 5;24:125. doi: 10.1186/s12931-023-02426-1 (PMC10161166; doi:10.1186/s12931-023-02426-1)
Supplement: Supplementary file 13 — Additional file 13. Discussion. [file 12931_2023_2426_MOESM13_ESM.docx]

Additional File 13.docx

Supplementary Discussion

Male sex, older age, and comorbidities, especially obesity, were associated with inflammation and tissue damage. We confirmed decreased lymphocyte counts and decreased production of effector T-cell cytokines in the CI, especially in the late phase of the disease.

Male sex and greater COVID-19 severity/mortality (1-6) have been related to increased expression of the SARS-CoV-2 angiotensin-converting enzyme 2 receptor and expression of the androgen hormone-controlled transmembrane serine protease 2 (TMPRSS2) gene, increasing the binding of SARS-CoV-2 protein S to angiotensin-converting enzyme 2 (7).

Advanced age has been linked to exhaustion and decline in native T-cell populations and diversity of the T-cell repertoire (8), contributing to the release of proinflammatory cytokines and decreased B-cell function (9).

Adipocytes produce cytokine-like hormones, called adipokines, which upregulate the production of proinflammatory cytokines and stimulate neutrophil chemotaxis and survival. This chronic inflammation leads to the inhibition of macrophage migration and activation and impaired formation of neutralisigng antibodies and memory T cells (10-12). Our results in patients with obesity, with increased leukocyte and neutrophil counts and TNF-α and IL-6 levels, and the finding in our multivariate study that obesity was the most important risk factor for a critical condition and ICU admission are consistent with the literature.

Some studies found high lymphocyte counts in BAL from Covid-19 patients. (13-15), whereas others have found lymphopenia (16). It is very likely that, more than the lymphocyte count, their effector activity or their depletion is critical to the evolution of the disease, as a recent study has reported. (17.). Another recent study found that the CD4 blood cell responses were even inversely correlated with absolute CD4 counts in patients with severe COVID-19 (18)

Lymphopenia in COVID-19 (19) is related to depletion and exhaustion of T cells (20-22), which occurs after inhibition of the innate interferon response and is due to the persistent antigenic activation of these cells, leading to a state of unresponsiveness (23,24). In our study, lymphocyte counts were very low and the N/L ratio was very high in the CI and non-survivors throughout the study, as was the case in other studies. There is evidence of lymphocyte recovery related to viral clearance and cure (25).

The correlation of the initial innate immune response cytokine TNF-α with T-cell-derived (CD4+) cytokines, IFN-γ and IL-17A, was significant, although this correlation decreased in the late inflammatory phase. In patients who reached critical status or died, there was no sustained increase in IFN-γ levels (as observed in the less severely ill patients [p<0.001]), coinciding with their persistent lymphopenia. Decreased production of IFNγ and type I IFN, as opposed to TNF-α, IL-6 and IL-8, has been reported in severe COVID patients (21,22). Our results confirm a decrease in lymphocyte counts and decreased production of effector T-cell cytokines in the most severely ill patients, especially in the late phase of disease progression (26).

Macrophages are activated by Pathogen-Associated Molecular Patterns (PAMPs) (such as viral RNA) and Damage-Associated Molecular Patterns (DAMPs), released after tissue damage. Innate immunity is activated, leading to antiviral gene expression (such as interferon) in cells in the neighbourhood of the infected cells. Macrophages also release cytokines such as TNF-α and IL-1β, which promote the release of other cytokines, such as IL-6, and recruit additional cells of innate and adaptive immunity, such as neutrophils and cytotoxic T cells. Thus, the macrophage-initiated cascade contributes to both viral control and tissue damage (27). In the late inflammatory phase of our critically ill patients and coinciding with severe and persistent lymphopenia, there was a spike in IL-6 levels, as well as higher levels of tissue damage markers (24). The presence of high circulating levels of IL-6 can activate signals in certain cells, such as endothelial cells, leading to systemic inflammation with additional expression of cytokines, which contribute to endothelium damage, vascular hyperpermeability, hypotension and pulmonary dysfunction (28,29), and directly affects the vascular endothelium favouring abnormal coagulation and increased permeability (30).

The systemic inflammatory response in COVID-19 is less robust than sepsis or cytokine storm syndrome (27). Cytokine storm is characterised by systemic hyperinflammation and multiorgan failure (29). In our study’s CI and non-survivors, only IL-6 showed a good correlation with cellular damage and a sustained increase in the late inflammatory phase. A more important role for cytokines in major cytokine storm could be expected.

The patients who received corticosteroids before day 17 of evolution had higher severity grades, and this treatment was an independent risk factor for pneumonia and for the extension of pulmonary infiltrates in >50% of lung fields, although it was not associated with higher mortality, as in a recent study (31). Although our results are consistent with studies reporting a relationship of severity with early administration of corticosteroids (32), the most severe patients probably received corticosteroids more frequently, so it is not possible to know which is the cause and which is the consequence of this relationship.
