# Supplementary material for: IL-6 and cfDNA monitoring throughout COVID-19 hospitalization are accurate markers of its outcomes
Source: Respir Res. 2023 May 5;24:125. doi: 10.1186/s12931-023-02426-1 (PMC10161166; doi:10.1186/s12931-023-02426-1)
Supplement: Supplementary file 14 — Additional file 14. References. [file 12931_2023_2426_MOESM14_ESM.docx]

Additional File 14.docx

Supplementary References

1. Gemmati D, Bramanti B, Serino ML, Secchiero P, Zauli G, Tisato V. COVID-19 and Individual Genetic Susceptibility/Receptivity: Role of ACE1/ACE2 Genes, Immunity, Inflammation and Coagulation. Might the Double X-chromosome in Females Be Protective against SARS-CoV-2 Compared to the Signgle X-Chromosome in Males? Int J Mol Sci, 2020; doi:10.3390/ijms21103474.

2. Cai H. Sex difference and smoking predisposition in patients with COVID-19. Lancet Respir. Med., 2020;8(4):e20.

3. Takahashi T, Ellingson MK, Wong P, Israelow B, Lucas C, Klein J, et al. Sex differences in immune responses that underlie COVID-19 disease outcomes. Nature, 2020; 588:7837:315–20.

4. Ssentongo P, Ssentongo AE, Heilbrunn ES, Ba DM, Chinchilli VM. Association of cardiovascular disease and 10 other pre-existing comorbidities with COVID-19 mortality: A systematic review and meta-analysis. PLoS ONE, 2020; doi: 10.1371/journal.pone.0238215.

5. Toth-Manikowski SM, Caldwell J, Joo M, Chen J, Meza N, Bruinius J, et al. Sex-related differences in mortality, acute kidney injury, and respiratory failure among critically ill patients with COVID-19. Medicine, 2021;100(50):E28302.

6. Viveiros A, Rasmuson J, Vu J, et al. Sex differences in COVID-19: candidate pathways, genetics of ACE2, and sex hormones. Am J Physiol Heart Circ Physiol, 2021;320(1):H296–304.

7. Amin MT, Fatema K, Arefin S, et al. Obesity, a major risk factor for immunity and severe outcomes of COVID-19. Biosci Rep, 2021. doi: 10.1097/MD.0000000000028302

8. Hazeldine J, Lord JM. Immunesenescence: A Predisposigng Risk Factor for the Development of COVID-19? Front Immunol, 2020;11.

9. Ademokun A, Wu YC, Dunn-Walters D. The ageing B cell population: composition and function. Biogerontology, 2010;11(2):125–37.

10. Taubenberger JK, Morens DM. 1918 Influenza: The mother of all pandemics. Emerg. Infect. Dis., 2006;12(1):15–22.

11. Morens DM, Fauci AS. The 1918 influenza pandemic: inSIhts for the 21st century. J Infect Dis, 2007;195(7):1018–28.

12. Demeulemeester F, de Punder K, van Heijningen M, van Doesburg F. Obesity as a Risk Factor for Severe COVID-19 and Complications: A Review. Cells, 2021;10(4):933. doi: 10.3390/cells10040933.

13. Voiriot G, Fajac A, Gibelin A, Parrot A, Fartouk M. Alveolar lymphocytosis with plasmacytosis in severe COVID-19. Respir Med Res. 2020; 78: 100784

14. Gelarden I, Nguyen J, Gao J, Chen Q, Morales-Nebreda L, Wunderink R, et al. Comprehensive evaluation of bronchoalveolar lavage from patients with severe COVID-19 and correlation with clinical outcomes. Hum Pathol. 2021 Jul;113:92-103. doi: 10.1016/j.humpath.2021.04.010.

15. Zaid Y, Doré É, Dubuc I, Archambault AS, Flamand O, Laviolette M, et al. Chemokines and eicosanoids fuel the hyperinflammation within the lungs of patients with severe COVID-19. J Allergy Clin Immunol. 2021 Aug;148(2):368-380. doi: 10.1016/j.humpath.2021.04.010.

16. Pandolfi L, Fossali T, Frangipane V, Bozzini S, Morosini M, D'Amato M, et al. Broncho-alveolar inflammation in COVID-19 patients: a correlation with clinical outcome. BMC Pulm Med. 2020 Nov 16;20(1):301. doi: 10.1016/j.humpath.2021.04.010.

17. Wauters E, Van Mol P, Garg AD, Jansen S, Van Herck Y, Vanderbeke L, et al. Discriminating mild from critical COVID-19 by innate and adaptive immune single-cell profiling of bronchoalveolar lavages. Cell Res. 2021 Mar;31(3):272-290. doi: 10.1016/j.humpath.2021.04.010.

18. Popescu I, Snyder ME, Iasella CJ, Hannan SJ, Koshy R, Burke R, et al. CD4+ T-Cell Dysfunction in Severe COVID-19 Disease Is Tumor Necrosis Factor-α/Tumor Necrosis Factor Receptor 1-Dependent. Am J Respir Crit Care Med. 2022 Jun 15;205(12):1403-1418. doi: 10.1016/j.humpath.2021.04.010.

19. Gao Y dong, Ding M, Dong X, Zhang JJ, Azkur AK, Azkur D, et al. Risk factors for severe and critically ill COVID-19 patients: A review. Allergy, 2021;76(2):428–55.

20. Feng Z, Diao B, Wang R, Wang G, Wang C, Tan Y, et al. The Novel Severe Acute Respiratory Syndrome Coronavirus 2 (SARS-CoV-2) Directly Decimates Human Spleens and Lymph Nodes. MedRxiv, 2020 Mar 31 [cited 2022 Apr 10]

21. Galani IE, Rovina N, Lampropoulou V, Triantafyllia V, Manioudaki M, Pavlos E, et al. Untuned antiviral immunity in COVID-19 revealed by temporal type I/III interferon patterns and flu comparison. Nat Immunol,2021;22(1):32–40.

22. Rodrigues PRS, Alrubayyi A, Pring E, Bart VMT, Jones R, Coveney C, et al. Innate immunology in COVID-19-a living review. Part II: dysregulated inflammation drives immunopathology. Oxf Open Immunol, 2020;1(1).

23. Moskophidis D, Lechner F, Pircher H, Zinkernagel RM. Virus persistence in acutely infected immunocompetent mice by exhaustion of antiviral cytotoxic effector T cells. Nature, 1993;362(6422):758–61.

24. Melms JC, Biermann J, Huang H, Wang Y, Nair A, Tagore S, et al. A molecular signgle-cell lung atlas of lethal COVID-19. Nature, 2021 ;595(7865):114–119.

25. Chen X, Ling J, Mo P, Zhang Y, Jiang Q, Ma Z, et al. Restoration of leukomonocyte counts is associated with viral clearance in COVID-19 hospitalized patients. MedRxiv, 2020 Mar 6 [cited 2022 Apr 11]. doi: https://doi.org/10.1101/2020.03.03.20030437

26. Chen G, Wu D, Guo W, Cao Y, Huang D, Wang H, et al. Clinical and immunological features of severe and moderate coronavirus disease 2019. The Journal of Clinical Investigation [Internet]. 2020 May 1 [cited 2022 Apr 11];130(5):2620–9. https://doi.org/10.1172/JCI137244.

27. Osuchowski MF, Winkler MS, Skirecki T, Cajander S, Shankar-Hari M, Lachmann G, et al. The COVID-19 puzzle: deciphering pathophysiology and phenotypes of a new disease entity. Lancet Respir Med, 2021;9(6):622–42.

28. Kang S, Tanaka T, Narazaki M, Kishimoto T. Targeting Interleukin-6 Signaling in Clinic. Immunity, 2019;50(4):1007–23.

29. Fajgenbaum DC, June CH. Cytokine Storm. NEJM, 2020;383(23):2255–73.

30. Kang S, Kishimoto T. Interplay between interleukin-6 Signaling and the vascular endothelium in cytokine storms. Exp Mol Med,2021;53(7):1116–23.

31. Benítez ID, de Batlle J, Torres G, González J, de Gonzalo-Calvo D, Targa ADS, et al. Prognostic implications of comorbidity patterns in critically ill COVID-19 patients: A multicenter, observational study. Lancet Reg Health Eur. 2022. DOI:https://doi.org/10.1016/j.lanepe.2022.100422.

32. Pérez de Llano LA, Golpe R, Pérez-Ortiz D, Menéndez R, España Yandiola PP, Artaraz A, et al. Early Initiation of Corticosteroids Might be Harmful in Patients Hospitalized With COVID-19 Pneumonia: A Multicenter Propensity Score Analysis. Arch Bronconeumol. 2022;58(3):281-283.
