# Supplementary material for: IL-6 and cfDNA monitoring throughout COVID-19 hospitalization are accurate markers of its outcomes
Source: Respir Res. 2023 May 5;24:125. doi: 10.1186/s12931-023-02426-1 (PMC10161166; doi:10.1186/s12931-023-02426-1)
Supplement: Supplementary file 15 — Additional file 15. Database. [file 12931_2023_2426_MOESM15_ESM.pdf]

TABLE 1 CASE GROUP **DATA OF AFFILIATION AND MEDICAL BACKGROUND**

patient	AGE	SEX	DEATH (1/0)	> 60 YEARS (1/0)	ARTERIAL HYPERTENSION (1/0)	Diabetes (1/0)	Obesity (1/0)	HEART DISEASE (1/0)	BRONCHOUMOPATHY (1/0)	LIVER DISEASE (1/0)	RENAL INSUFFICIENCY (1/0)	DEMENTIA (1/0)	DYSLIPEMIA (1/0)
1	57	F	0	0	0	0	0	0	0	0	0	0	1
2	52	F	0	0	0	0	0	0	0	0	0	0	0
3	67	F	1	1	1	0	1	0	1	0	0	0	1
4	58	F	0	0	1	0	0	0	0	0	0	0	0
5	63	F	0	1	0	0	0	0	0	0	0	0	1
6	49	M	0	0	0	0	0	0	0	0	0	0	0
7	54	F	0	0	0	0	0	0	1	0	0	0	0
8	55	M	0	0	0	1	0	0	0	0	0	0	1
9	48	M	0	0	1	0	0	0	0	0	0	0	0
10	70	M	0	1	0	1	0	0	0	0	0	0	0
11	75	M	1	1	1	0	0	0	1	0	0	0	0
12	72	F	1	1	1	0	1	1	0	0	0	0	0
13	62	M	0	1	0	0	0	1	0	0	0	0	0
14	88	F	0	1	1	1	0	0	0	0	0	0	1
15	76	F	0	1	1	0	0	0	0	0	0	1	1
16	88	F	1	1	1	0	0	0	1	0	0	0	0
17	76	M	0	1	0	0	0	0	0	0	0	0	0
18	86	M	0	1	0	1	0	1	1	0	0	1	0
19	26	F	0	0	0	0	0	0	0	0	0	0	0
20	74	F	0	0	0	0	0	0	0	0	0	1	0
21	56	F	0	0	1	1	0	1	1	0	0	0	1
22	81	F	0	1	0	0	0	0	0	0	0	1	1
23	33	F	0	0	0	0	0	0	0	0	0	0	1
24	77	M	1	1	1	0	0	0	1	0	0	0	0
25	79	M	0	1	0	1	0	0	0	0	0	0	0
26	72	F	0	1	0	0	0	0	0	0	0	0	0
27	68	M	0	1	1	0	0	0	0	0	1	0	0
28	78	M	1	1	1	1	0	1	1	0	0	0	0
29	35	F	0	0	0	0	0	0	0	0	0	0	0
30	47	F	0	0	0	0	0	0	0	0	0	0	0
31	46	F	0	0	0	0	0	0	0	0	0	0	0
32	66	M	0	1	1	0	0	1	0	0	1	1	0
33	75	F	0	1	1	1	1	0	1	0	0	0	1
34	71	F	0	1	1	0	0	0	0	0	0	0	0
35	63	F	0	1	0	0	0	0	0	0	0	0	0
36	69	F	0	1	0	0	0	0	0	0	0	1	0
37	55	F	0	0	0	1	0	0	0	0	0	0	0
38	64	M	0	1	1	1	0	0	0	0	0	0	0
39	61	M	0	1	0	0	0	1	0	0	1	0	0
40	51	F	0	0	0	0	0	0	0	0	0	0	0
41	53	M	0	0	0	0	0	0	0	0	0	0	0
42	48	M	0	0	0	0	0	0	0	0	0	0	0
43	79	M	1	1	0	1	0	0	0	0	0	1	0
44	77	M	0	1	1	1	0	0	0	0	0	0	0
45	84	F	0	1	1	1	1	0	0	0	0	0	0
46	83	M	0	1	1	0	0	0	0	0	0	1	1
47	68	F	1	1	1	0	0	0	0	0	0	0	0
48	33	F	0	0	0	0	0	0	0	0	0	0	0
49	76	F	0	1	1	0	0	1	0	0	0	1	0
50	85	F	0	1	0	0	0	1	1	0	0	1	0
51	78	M	0	1	1	1	0	0	0	0	0	1	0
52	79	F	1	1	1	0	0	0	0	0	0	1	0
53	77	M	0	1	1	1	0	1	0	0	0	1	1
54	72	M	0	1	1	1	0	1	0	0	0	0	0
55	82	F	0	1	1	1	1	0	0	0	1	0	0
56	56	F	0	0	0	0	0	0	1	0	0	0	0
57	43	M	0	0	0	1	0	0	0	1	0	0	1
58	42	F	0	0	0	0	0	0	0	0	0	0	0
59	68	M	1	1	1	0	0	0	0	0	0	0	1
60	59	F	0	0	1	1	1	0	0	0	0	0	1
61	85	F	0	1	1	0	0	0	0	0	0	1	0
62	37	M	0	0	0	0	0	0	0	0	0	0	0
63	87	M	1	1	1	0	0	0	1	0	1	1	0
64	86	M	0	1	1	1	0	0	0	0	1	0	0
65	71	M	0	1	1	1	0	0	0	0	0	0	1
66	87	M	0	1	0	0	0	1	1	0	0	1	0
67	36	M	0	0	0	0	0	0	0	0	0	0	0
68	40	F	0	0	0	0	0	0	0	0	0	0	0
69	80	F	0	1	1	1	0	0	0	1	0	0	0
70	60	F	0	1	1	0	0	0	0	0	0	0	0
71	76	M	0	1	1	0	0	0	0	0	0	0	0
72	87	F	0	1	1	1	0	0	0	0	0	0	0
73	82	F	0	1	0	1	0	0	1	0	0	1	0
74	49	F	0	0	0	0	0	0	0	0	0	0	0
75	41	F	0	0	0	0	1	0	0	0	0	0	0
76	80	F	0	1	1	0	0	0	1	0	0	1	0
77	83	M	0	1	1	1	0	0	0	0	1	0	0
78	86	F	1	1	0	0	0	0	0	0	1	0	0
79	29	F	0	0	0	0	0	0	0	0	0	0	0
80	93	M	1	1	1	0	0	1	0	0	0	0	0
81	58	M	0	0	1	0	0	0	0	0	0	0	0
82	76	M	1	1	1	0	1	0	0	0	0	0	1
83	56	M	1	0	0	0	0	0	0	0	0	0	0
84	53	F	1	0	0	0	1	0	0	0	0	0	0

85	71	M	0	1	0	0	0	0	0	0	0	0	0
86	62	M	0	1	1	0	1	0	0	0	0	0	0
87	68	M	0	1	1	1	1	0	0	0	0	0	0
88	67	M	1	1	1	0	0	0	0	1	0	0	0
89	71	F	1	1	1	1	1	0	1	0	0	0	0
90	71	F	0	1	0	0	0	0	0	0	0	0	0
91	73	M	0	1	0	0	0	1	0	0	0	0	0
92	47	F	0	0	0	1	1	0	0	0	0	0	0
93	77	M	1	1	0	0	0	0	0	0	0	0	0

TABLE 2 CASE GROUP **CLINICAL SITUATION DURING THE STUDY (at the moments of greatest severity)**

patient	Fever >38º (1/0)	Hypotension (<90mmHg SBP)	Tachycardia >100 (1/0)_	Tachypnea (>25) (1/0)	Confusión (1/0)	basal SaO2	inspiratory fraction of O2	PaO2/FiO2	SaO2/FiO2	Noninvasive mechanical ventilation (1/0)	Invasive mechanical ventilation (1/0)	high flow O2 (1/0)	vasopressors (1/0)	ECMO (1/0)	Myocarditis (1/0)	Encephalitis (1/0)	Failure of 1 organ (1/0)	Multi-organic failure >1org (1/0)	Sepsis (1/0)	Shock (1/0)	Severity (1: mild-moderate; 2: serius; 3: critical)	Ordinal Scale
1	1	0	0	1	0	80	0,6	136	160	0	0	0	0	0	0	0	0	0	0	0	1	4
2	0	0	0	0	0	99	0,21	628	471	0	0	0	0	0	0	0	0	0	0	0	1	4
3	0	0	0	1	0		1	55	88	0	0	1	0	0	0	0	0	0	0	0	3	8
4	0	0	0	0	0	90	0,36	227	267	0	0	0	0	0	0	0	0	0	0	0	2	5
5	0	0	0	0	0	94	0,21	337	448	0	0	0	0	0	0	0	0	0	0	0	1	4
6	0	0	0	0	0	96	0,21	390	457	0	0	0	0	0	0	0	0	0	0	0	1	4
7	0	0	0	0	0	99	0,21	628	471	0	0	0	0	0	0	0	0	0	0	0	1	4
8	0	0	0	0	0	83	1	71	111	0	0	0	0	0	0	0	0	0	0	0	3	5
9	0	0	0	0	0	96	0,21	390	457	0	0	0	0	0	0	0	0	0	0	0	2	4
10	0	0	1	1	0	92	0,21	304	438	0	0	0	0	0	0	0	0	0	0	0	2	4
11	1	1	1	1	0	90	1		63	0	0	1	0	0	0	0	1	0	0	o	3	8
12	0	0	0	1	1	87	0,8	102	120	0	0	0	0	0	0	0	0	0	0	0	3	8
13	0	1	0	0	0	95	0,21	360	452	0	0	0	0	0	0	0	0	0	0	0	2	4
14	0	0	0	0	0	96	0,21	390	457	0	0	0	0	0	0	0	0	0	0	0	1	4
15	0	0	0	0	0	90	0,28	323	346	0	0	0	0	0	0	0	0	0	0	0	1	5
16	0	0	0	1	1		1	91	97	0	0	0	0	0	0	0	1	0	0	0	3	8
17	0	0	0	1	0	87	0,28	372	350	0	0	0	0	0	0	0	0	0	0	0	2	5
18	0	0	0	1	0	89	0,8	101	134	0	0	0	0	0	0	0	0	0	0	0	2	5
19	1	1	1	1	1		1		71	0	0	1	0	0	0	0	1	0	0	0	3	8
20	0	1	0	0	0	95	0,36	496	467	0	0	0	0	0	0	0	0	0	0	0	2	5
21	0	0	0	0	0	97	0,21	431	462	0	0	0	0	0	0	0	0	0	0	0	1	3
22	1	0	0	0	1	85	0,36	227	267	0	0	0	0	0	0	0	0	0	0	0	2	5
23	1	0	0	0	0	96	0,21	390	457	0	0	0	0	0	0	0	0	0	0	0	1	4
24	0	1	0	0	1		1		78	0	0	0	0	0	0						3	8
25	1	0	0	0	0	88	0,4	168	233	0	0	0	0	0	0	0	1	0	0	0	3	5
26	0	0	0	0	0	95	0,21	360	452	0	0	0	0	0	0	0	0	0	0	0	1	4
27	0	0	0	0	0	96	0,21	390	457	0	0	0	0	0	0	0	0	0	0	0	1	4
28	1	0	0	0	0	70	1	51	86	1	0	1	0	0	0	0	0	1	0	0	3	8
29	0	0	0	0	0	100	0,21	628	471	0	0	0	0	0	0	0	0	0	0	0	1	4
30	0	0	0	0	0	97	0,21	431	462	0	0	0	0	0	0	0	0	0	0	0	1	4
31	0	0	0	1	0	93	0,21	209	291	0	0	0	0	0	0	0	0	0	0	0	1	5
32	0	0	0	0	1	90	0,36	197	261	0	0	0	0	0	0	0	0	0	0	0	2	5
33	0	0	0	0	0	90	0,28	253	336	0	0	0	0	0	0	0	0	0	0	0	2	5
34	0	0	0	0	0	98	0,21	390	457	0	0	0	0	0	0	0	0	0	0	0	1	4
35	0	0	0	0	0	99	0,21	628	471	0	0	0	0	0	0	0	0	0	0	0	1	4
36	0	0	0	0	0	95	0,21	360	452	0	0	0	0	0	0	0	0	0	0	0	1	4
37	0	0	1	0	0	95	0,21	360	452	0	0	0	0	0	0	0	0	0	0	0	1	4
38	0	0	0	1	0	87	0,8	89	118	0	0	0	0	0	0	0	0	0	0	0	1	5
39	1	0	0	0	0	99	0,21	628	471	0	0	0	0	0	0	0	0	0	0	0	1	4
40	0	0	0	0	0	98	0,21	390	457	0	0	0	0	0	0	0	0	0	0	0	1	4
41	1	0	0	0	0	93	0,21	123	442	0	0	0	0	0	0	0	0	0	0	0	1	4
42	1	0	0	0	0	97	0,21	431	462	0	0	0	0	0	0	0	0	0	0	0	1	4
43	1	0	0	1	0	85	0,4	168	233	0	0	0	0	0	0	0	0	0	0	0	3	8
44	1	0	0	0	0	98	0,21	390	457	0	0	0	0	0	0	0	0	0	0	0	1	4
45	1	0	1	0	0	87	0,8	102	120	0	0	0	0	0	0	0	0	0	0	0	2	4
46	0	0	0	0	0	88	0,32	221	294	0	0	0	0	0	0	0	0	0	0	0	2	4
47	0	0	1	1	1		1	50	85	1	0	1	0	0	0	0	0	0	0	0	3	8
48	0	0	0	0	0	99	0,21	628	471	0	0	0	0	0	0	0	0	0	0	0	1	4
49	0	0	0	0	0		0,28	471	354	0	0	0	0	0	0	0	0	0	0	0	1	5
50	0	0	0	0	0	90	0,21	279	429	0	0	0	0	0	0	0	1	0	0	0	1	4
51	1	0	0	0	0	91	0,21	291	433	0	0	0	0	0	0	0	1	0	0	0	1	4
52	1	0	0	1	1		0,7	130	87	0	0	0	0	0	0	0	1	0	0	0	3	8
53	1	0	0	0	0		1	50	85	0	0	0	0	0	0	0	0	0	0	0	2	5
54	0	0	0	0	1	87	0,28	270	339	0	0	0	0	0	0	0	0	0	0	0	2	5
55	0	0	0	0	0	94	0,21	337	448	0	0	0	0	0	0	0	0	0	0	0	1	4
56	0	0	0	0	0	93	0,21	319	443	0	0	0	0	0	0	0	0	0	0	0	1	4
57	0	0	0	0	0	97	0,21	431	462	0	0	0	0	0	0	0	0	0	0	0	1	4
58	0	1	0	0	0	93	0,21	319	443	0	0	0	0	0	0	0	0	0	0	0	1	4
59	1	0	0	1	1		1	57	89	0	0	0	0	0	0	0	0	0	0	0	3	8
60	0	0	1	0	0	91	0,28	270	339	0	0	0	0	0	0	0	0	0	0	0	1	5
61	0	0	0	0	0	95	0,21	360	452	0	0	0	0	0	0	0	0	0	0	0	1	4
62	0	0	0	0	0	98	0,21	496	467	0	0	0	0	0	0	0	0	0	0	0	1	4
63	1	1	1	1	1		0,36	264	330	0	0	0	0	0	0	0	0	0	0	0	2	5
64	0	0	0	0	1	93	0,21	319	443	0	0	0	0	0	0	0	1	0	0	0	2	4
65	0	0	0	0	0	98	0,21	496	467	0	0	0	0	0	0	0	0	0	0	0	1	4
66	0	0	0	0	1	86	0,36	264	330	0	0	0	0	0	0	0	1	0	0	0	1	5
67	0	0	0	0	0	99	0,21	471	460	0	0	0	0	0	0	0	0	0	0	0	1	4
68	0	1	0	0	0	99	0,21	471	460	0	0	0	0	0	0	0	0	0	0	0	1	4
69	0	0	0	0	0	97	0,21	431	462	0	0	0	0	0	0	0	0	0	0	0	1	4
70	0	0	0	0	0		0,32	191	284	0	0	0	0	0	0	0	0	0	0	0	1	5
71	0	0	0	0	0	97	0,21	431	462	0	0	0	0	0	0	0	0	0	0	0	1	5
72	0	1	0	0	1	94	0,21	337	448	0	0	0	0	0	0	0	0	0	0	0	1	5
73	0	0	0	0	0	95	0,21	360	452	0	0	0	0	0	0	0	0	0	0	0	1	3
74	0	0	0	0	0	99	0,21	628	471	0	0	0	0	0	0	0	0	0	0	0	1	3
75	0	0	0	0	0	96	0,21	390	457	0	0	0	0	0	0	0	0	0	0	0	1	3
76	0	0	0	0	0	90	0,28	319	443	0	0	0	0	0	0	0	1	0	0	0	1	5
77	1	0	0	0	0	96	0,21	390	457	0	0	0	0	0	0	0	0	0	0	0	1	3
78	1	0	1	1	1		1	67	93	0	0	0	0	0	0	0	1	0	0	0	3	8
79	1	0	0	0	0	99	0,21	628	471	0	0	0	0	0	0	0	0	0	0	0	1	3
80	0	0	0	1	0	74	1	71	94	0	0	0	0	0	0	0	1	0	0	0	3	8
81	0	0	0	0	0	96	0,21	390	457	0	0	0	0	0	0	0	0	0	0	0	1	4
82	1	1	1	1	0	85	1	110 (con VM)		0	1	1	0	0	0	0	1	1	1	0	3	8
83	1	1	1	1	1	50	1	60 (con VM)		0	1	0	1	0	0	0	1	1	1	1	3	8
84	1	0	0	1	0	80	1	80 (con VM)		0	1	1	1	1	0	0	1	0	0	0	3	8
85	1	1	1	1	1	80	1	90 (con VM)		1	1	1	0	0	0	0	1	0	1	0	3	8
86	0	0	0	1	0	85	1	60 (con alto flujo)		0	0	1	0	0	0	0	1	0	0	0	3	6
87	1	0	1	1	0	85	1	75 (con alto flujo)		0	0	1	0	0	0	0	1	0	0	0	3	7
88	0	0	0	1	1	93	1	113 (con VM)	93	0	1	1	0	0	1	0	1	0	0	0	3	8
89	0	0	1	1	1	85	1	88 (con VM)	88	0	1	1	1	0	0	0	1	0	0	0	3	8
90	0	0	0	0	0	95	0,21	150		0	1	1	1	0	0	0	1	0	0	0	3	7
91	0	1	0	0	0	94	0,21	292		0	1	1	1	0	0	0	1	0	0	0	3	7
92	0	1	1	0	1	88	0,28	197		0	1	0	1	0	0	0	1	0	0	0	3	7
93	1	1	1	1	0	88	0,32	164	180	0	1	0	1	0	0	0	1	1	1	1	3	8

	NEUMONIA (1/0)	Pulmonary infiltrates in >50% of radiological fields	ARDS diffuse pattern
1	1	1	0
2	1	0	0
3	1	0	0
4	1	1	0
5	1	0	0
6	1	0	0
7	1	0	0
8	1	1	0
9	1	0	0
10	1	0	0
11	1	1	0
12	1	1	0
13	1	0	0
14	1	1	0
15	1	0	0
16	1	0	0
17	1	1	0
18	1	0	0
19	1	0	0
20	1	0	0
21	0	0	0
22	0	0	0
23	1	0	0
24	0	0	0
25	1	0	0
26	1	0	0
27	1	1	0
28	1	0	0
29	1	1	0
30	1	0	0
31	1	0	0
32	1	0	0
33	1	0	0
34	1	0	0
35	1	0	0
36	1	0	0
37	1	0	0
38	1	0	0
39	1	0	0
40	1	0	0
41	1	0	0
42	1	1	0
43	1	0	0
44	1	0	0
45	1	0	0
46	1	0	0
47	1	0	0

48	1	0	0
49	1	0	0
50	0	0	0
51	1	0	0
52	1	0	0
53	1	0	0
54	0	0	0
55	1	0	0
56	1	0	0
57	1	0	0
58	1	0	0
59	1	0	0
60	0	0	0
61	0	0	0
62	1	0	0
63	1	0	0
64	1	0	0
65	1	0	0
66	0	0	0
67	0	0	0
68	1	0	0
69	1	0	0
70	1	0	0
71	1	0	0
72	0	0	0
73	1	0	0
74	1	0	0
75	1	0	0
76	0	0	0
77	0	0	0
78	1	0	0
79	1	0	0
80	0	0	0
81	1	0	0
82	1	0	0
83	1	1	0
84	1	1	1
85	1	1	0
86	1	1	0
87	1	1	0
88	1	1	1
89	1	1	1
90	1	0	0
91	1	0	0
92	1	1	0
93	1	1	0

	Test rápido de anticuerpos (1/0)	PCR SARS-COV-2 (0/1)
1	0	1
2	0	1
3	0	1
4	0	1
5	0	1
6	0	1
7	0	1
8	0	1
9	0	1
10	0	1
11	0	1
12	0	1
13	0	1
14	0	1
15	0	1
16	0	1
17	0	1
18	0	1
19	0	1
20	0	1
21	0	1
22	0	1
23	0	1
24	0	1
25	0	1
26	0	1
27	0	1
28	0	1
29	0	1
30	1	1
31	0	1
32	0	0
33	0	1
34	0	1
35	0	1
36	0	1
37	0	1
38	0	1
39	0	0
40	1	0
41	0	1
42	0	1
43	0	1
44	0	1
45	0	1
46	0	1
47	0	1
48	0	1

49	0	1
50	0	1
51	0	1
52	1	1
53	0	1
54	0	1
55	0	1
56	0	1
57		
58	0	1
59	0	1
60	0	1
61	0	1
62	0	1
63		
64		
65	0	1
66	0	1
67		
68	0	1
69	0	1
70	0	1
71	0	1
72	0	1
73	0	1
74	0	1
75	1	1
76	0	1
77	0	1
78	0	1
79	0	1
80	0	1
81	0	1
82	0	1
83	0	1
84	0	1
85	0	1
86	0	1
87	0	1
88		1
89		1
90	0	1
91	0	1
92	0	1
93	0	1

	Tocilizumab (0/1)	CORTICOIDS (0/1)	Remdesivir (0/1)	VASOACTIVE DRUGS (0/1)
1	0	0	0	0
2	0	0	0	0
3	0	0	0	0
4	0	0	0	0
5	0	0	0	0
6	0	0	0	0
7	0	0	0	0
8	0	1	0	0
9	0	0	0	0
10	0	1	0	0
11	0			0
12	1	1	0	0
13	0	0	0	0
14	0	0	0	0
15	0	0	0	0
16				
17	0	0	0	0
18	0	0	0	0
19	0			0
20	0	0	0	0
21	0	0	0	0
22	0	1		0
23	0	0	0	0
24	0	0	0	0
25	0	0	0	0
26	0	0	0	0
27	0	0	0	0
28	1	1	0	0
29	0	0	0	0
30	0	0	0	0
31	0	0	0	0
32	0	0	0	0
33	1	0	0	0
34	0	0	0	0
35	0	0	0	0
36	0	0	0	0
37	0	1	0	0
38	0	1	0	0
39	0	0	0	0
40	0	0	0	0
41	0	1	0	0
42	0	1	0	0
43	0	0	0	0
44	0	0	0	0
45	0	1	0	0
46	0	0	0	0
47	0	1	0	0
48	0	0	0	0
49	0	0	0	0

50	0	0	0	0
51	0	0	0	0
52	0	1	0	0
53	0	1	0	0
54	0	0	0	0
55	0	1	0	0
56	0	0	0	0
57	0	0	0	0
58	0	0	0	0
59	0	0	0	0
60	0	0	0	0
61	0	1	0	0
62	0	0		
63	0	1	0	0
64	0	0		
65	0	0	0	0
66	0	0		
67	0	0	0	0
68	0	0	0	0
69	0	0	0	0
70	0	0	0	0
71	0	0	0	0
72	0	0	0	0
73	0	0	0	0
74	0	0	0	0
75	0	0	0	0
76	0	0	0	0
77	0	0	0	0
78	0	0	0	0
79	0	0	0	0
80	0	0	0	0
81	0	0	0	0
82	1	1	0	0
83	0	1	0	0
84	0	1	1	0
85	1	1	1	0
86	0	1	0	0
87	1	1	0	0
88	0	1	1	0
89	0	0	0	0
90	0	1	0	1
91	0	1	0	1
92	0	1	1	1
93	0	1	0	1

PATIENT CASE Nº	DETERMINATION	Group	TNF-A	IL-8	IL-1B	IFN G	IL-17A	P SEL	G-CSF	IL6	LDH	cfDNA
1	1	Case	32,37	29,34	42,15	119,97	10,79	29194,78	269,35	147,80	427	17954,78
	2	Case	34,83	43,56	44,03	116,20	14,95	38381,25	131,66	5,72	435	10426,13
	3	Case	40,96	49,15	52,52	149,47	14,95	79931,17	126,03	2,53	348	10498,02
	4	Case	42,79	46,72	59,15	153,09	22,80	131368,06	136,15	1,63	325	13966,13
2	1	Case	37,84	35,06	55,23	130,64	18,48	83243,70	130,47	13,03	247	8111,65
	2	Case	47,13	55,52	68,55	178,09	23,90	65724,66	150,75	3,49	175	8890,70
3	1	Case	31,76	20,50	44,97	93,05	10,79	24599,97	161,75	14,18		24849,87
	2	Case	38,75	28,52	58,71	134,40	14,73	26842,54	206,57	11,36	503	22379,83
	3	Case	61,74	272,92	68,66	134,86	18,94	46560,85	322,72	453,30		55526,97
4	1	Case	34,83	56,50	46,85	119,97	14,95	49253,98	243,55	4,85	273	12587,54
	2	Case	54,62	285,27	76,97	206,31	32,56	101694,90	165,22	2,78	157	7008,59
5	1	Case	20,31	42,11	38,40	100,87	6,36	24297,92	106,74	34,21	239	4411,73
	2	Case	28,57	80,94	54,50	144,28	19,40	32160,86	136,15	52,94	250	4443,70
	3	Case	37,07	398,52	72,76	182,83	28,28	56126,55	153,66	14,04	213	1607,95
6	1	Case	38,40	50,02	56.23	148.52	19,35	56128,24	142.84	6,40	211	7848,16
	2	Case	47,97	55,19	71,36	178,09	23,90	58484,80	200,91	21,87	223	
7	1	Case	44,63	41,93	71,36	173,32	23,90	72053,31	162,33	<1,5		9043,73
	2	Case	41,28	38,41	64,34	175,71	28,28	67189,32	156,55	<1,5	203	10130,62
	3	Case	52,96	39,69	78,37	215,59	32,56	92798,90	179,56	<1,5	197	9735,39
9	1	Case	24,97	29,82	44,03	116,20	10,79	44116,88	99,87	2,94	207	4006,98
	2	Case	37,91	43,85	73,46	180,46	19,40	66682,74	139,08	5,42	272	4989,31
10	1	Case	50,47	55,84	61,53	182,83	28,28	56126,55	146,39	23,16	254	7410,11
11	1	Case	39,28	54,11	54,04	130,64	18,48	53046,98	135,34	168,70	254	7684,83
	2	Case	36,06	42,35	55,35	134,86	18,94	50992,67	122,64	<1,5	270	4643,67
12	1	Case	39,12	52,82	53,46	147,66	22,80	91757,65	124,90	35,25	390	4257,12
	2	Case	36,36	69,37	53,46	156,69	18,94	81582,55	124,90	57,27	586	7192,02
13	1	Case	34,24	56,48	39,43	97,91	15,67	36997,58	118,08	127,60	707	13123,42
	2	Case	45,35	61,23	56,40	126,67	24,16	74090,07	148,51	4,58		22759,75
14	1	Case	40,35	738,36	53,45	122,66	24,16	57944,18	121,83	25,76	211	7940,04
	2	Case	31,35	36,88	41,93	102,13	15,67	24290,30	120,58	33,57		
15	1	Case	35,44	25,27	42,62	112,40	10,79	48413,23	122,64	28,91	258	4258,47
	2	Case	45,88	54,21	55,90	163,73	14,73	50830,41	136,15	9,61		8336,18
16	1	Case	32,80	213,03	35,60	110,45	21,31	46475,05	375,35	160,70	188	3522,29
	2	Case	32,80	237,13	36,89	110,45	18,48	47419,69	132,92	11,19		4631,13
17	1	Case	39,43	35,10	53,46	154,89	18,94	73990,72	175,00	86,92	445	6290,10
	2	Case	39,12	46,72	53,46	134,86	14,95	65380,64	133,90	<1,5	389	20138,19
18	1	Case	48,80	416,53	86,79	210,96	23,90	88280,94	166,66	39,23	330	18160,42
	2	Case	45,46	81,63	76,97	175,71	28,28	62893,70	159,45	2,03	274	9735,39
	3	Case	39,60	48,04	65,74	158,91	14,73	55476,85	156,55	5,85	229	4652,92
19	1	Case	47,97	50,31	65,74	154,06	23,90	61113,01	159,45	3,00	170	1052,84
	2	Case	44,63	45,78	72,06	185,20	23,90	68710,71	147,85	< 1,5	159	5181,50

20	1	Case	36,40	52,01	48,06	126,67	21,31	44798,19	2583,68	927,40	402	8261,25
	2	Case	30,62	31,14	46,85	118,62	21,31	54401,74	128,02	5,97	324	14861,48
	3	Case	36,40	77,32	54,04	134,60	24,16	72520,03	135,34	3,49	162	8120,45
21	1	Case	50,33	44,95	61,08	150,18	21,31	75750,99	140,17	27,91		6122,78
22	1	Case	34,83	34,86	44,50	100,87	6,36	20799,57	136,15	44,41	394	4789,79
	2	Case	37,28	52,33	44,03	89,09	10,79	20211,76	109,02	51,33	410	14089,77
23	1	Case	26,21	56,25	51,57	131,17	14,95	58817,90	120,38	4,37	250	3833,83
	2	Case	34,53	77,53	69,95	192,27	23,90	55495,05	152,21	< 1,5	202	2942,22
24	1	Case	43,79	72,78	57,31	154,06	14,73	42766,54	170,97	103,50	421	9150,35
25	1	Case	42,22	51.52	54,34	139,98	19,20	43674,28	169,37	44,99	238	
	2	Case	47,13	48,36	58,71	192,27	19,40	44192,99	196,65	104,20	374	4627,63
	3	Case	52,88	59,95	61,05	156,69	18,94	618539,08	162,86	449,40	312	8788,71
	4	Case	31,76	37,03	41,21	100,87	1,28	35775,83	104,45	4,15		
26	1	Case	46,54	43,85	51,24	145,28	22,86	62455,58	137,83	39,25	410	
	2	Case	47,28	46,24	56,30	156,31	23,24	63287,94	140,24	26,54	426	3272,35
	3	Case	48,63	46,56	59,59	157,67	24,97	64125,57	149,42	< 1,5	239	
27	1	Case	39,12	47,45	47,80	134,86	10,79	48322,14	219,77	INSUF	301	14901,08
	2	Case	36,67	55,02	41,21	108,58	6,36	34864,50	184,88	86,94	371	27337,79
	3	Case	52,88	59,95	61,05	156,69	18,94	618539,08	162,86	449,40	306	16335,53
28	1	Case	67,86	76,85	69,95	192,27	19,40	59364,14	159,45	Insuf	147	22670,38
	2	Case	42,53	77,53	54,50	134,40	14,73	35289,61	254,29	265,70	181	9000,92
29	1	Case	33,26	26,29	51,68	121,88	19,40	35619,89	121,38	<1,5	133	2698,11
	2	Case	38,32	35,36	56,32	132,22	26,61	48745,40	129,14	<1,5	143	2838,19
30	1	Case	45,46	40,01	64,34	166,14	23,90	52209,41	153,66	<1,5	224	
	2	Case									211	9657,08
	3	Case									211	
31	1	Case	44,02	49,89	48,74	134,86	10,79	71354,79	147,32	38,24	162	1908,54
	2	Case	50,88	61,10	61,53	163,73	14,73	54476,04	159,45	33,45	171	5593,08
32	1	Case	41,57	53,80	45,91	131,17	3,96	47204,62	147,32	53,68	407	21618,00
	2	Case	42,55	127,48	62,54	175,23	26,42	69474,22	148,90	83,83	368	
	3	Case	50,47	132,79	69,95	187,56	28,28	72554,97	150,75	4,87	321	7142,31
33	1	Case	57,94	58,80	68,55	182,83	23,90	69668,40	153,66	10,26	166	1906,50
	2	Case	60,42	168,27	69,95	187,56	19,40	65767,90	127,31	17,76	163	8210,53
	3	Case	53,79	102,29	74,16	182,83	23,90	69142,23	139,08	2,99	260	3284,85
	4	Case	66,21	304,51	85,39	210,96	28,28	116107,83	158,00	2,66	170	7446,34
34	1	Case	34,24	26,68	46,85	114,55	15,67	58060,15	120,58	6,33	132	2796,27
	2	Case	40,44	51,61	61,53	168,54	23,90	52942,90	150,75	<1,5	136	4446,77
35	1	Case	36,06	28,38	52,04	145,84	14,95	69523,08	118,12	5,60	208	4353,92
	2	Case									169	
36	1	Case	29,91	31,74	47,80	119,97	8,62	44148,78	118,12	28,70	356	20093,26
	2	Case	40,96	90,07	62,95	178,02	18,94	160857,23	140,62	36,59		10014,11
37	1	Case	37,84	33,76	56,40	146,32	24,16	63962,54	148,51	78,17	175	4226,32
	2	Case	46,93	42,51	66,69	179,26	28,25	68689,74	158,26	4,23	168	3300,47

38	1	Case	28,99	33,66	43,09	266,62	10,79	38953,27	99,87	58,54	557	18794,25
	2	Case	41,70	83,01	66,44	391,13	23,90	58561,58	142,01	2,17	167	2051,09
39	1	Case	46,46	119,28	25,37	96,98	6,36	27030,03	495,97	161,10	167	7937,48
	2	Case	31,76	110,03	32,80	140,37	14,95	43454,51	118,12	4,96	146	5444,58
40	1	Case	35,44	77,89	40,28	134,86	14,95	75279,56	102,16	<1,5	167	10807,12
	2	Case									146	
41	1	Case	27,72	39,37	64,34	141,82	19,40	29240,97	243,11	<1,5	150	8346,69
	2	Case	36,22	66,41	74,16	208,64	19,40	53580,88	153,66	< 1,5	206	6286,99
42	1	Case	36,06	27,18	43,09	188,53	14,95	59040,55	120,38	<1,5	281	686,42
	2	Case									167	
43	1	Case	28,68	19,30	40,28	98,93	12,89	28810,38	109,02	39,39	232	4036,78
	2	Case									254	
44	1	Case	35,14	56,74	41,21	96,98	6,36	32887,09	102,16	58,79	306	6866,42
	2	Case	44,63	81,97	58,01	144,28	19,40	44273,51	136,15	29,47	320	6886,19
	3	Case	50,88	65,74	72,76	178,09	23,90	70640,16	147,85	< 1,5	199	3405,09
45	1	Case	53,79	145,41	79,08	182,83	28,28	72819,23	153,66	10,06	224	7057,69
	2	Case	49,62	104,84	51,66	122,66	18,48	51576,01	124,32	25,30	281	22830,15
	3	Case	49,63	107,31	68,55	196,97	23,90	56384,84	134,68	36,56	300	8628,74
46	1	Case	41,93	102,13	15,67	24290,30	120,58	33,57	0,943	0,804	255	5427,56
	2	Case									269	
47	1	Case	42,53	42,57	65,74	168,54	19,40	47829,25	153,66	3,40	417	14108,31
	2	Case	44,28	298,74	72,54	173,00	24,16	94710,05	142,57	4,26	513	20203,26
48	1	Case	27,44	19,54	35,60	93,05	6,36	27283,23	102,16	6,04	178	5706,50
	2	Case									174	
49	1	Case	47,13	113,85	50,27	134,40	19,40	35469,43	227,70	110,30	260	9560,83
	2	Case	29,91	27,42	39,34	93,05	10,79	30851,33	106,74	14,21	239	3120,06
	3	Case	41,28	40,65	60,12	139,36	19,40	47797,75	133,21	22,92	257	1484,92
50	1	Case	39,43	66,88	48,74	138,54	10,79	69037,47	132,78	135,60	353	13694,12
	2	Case	55,45	65,08	81,18	215,59	32,56	102359,14	183,85	32,21	194	7525,38
51	1	Case	39,60	50,31	58,71	163,73	19,40	45280,39	144,93	22,03	151	1797,81
	2	Case	37,84	42,09	51,66	122,66	18,48	69157,08	152,06	34,12	219	9101,68
	3	Case	40,44	126,03	55,90	168,54	19,40	65422,83	153,66	15,73	182	9759,76
52	1	Case	26,21	58,22	36,53	72,90	6,36	18364,57	109,02	31,73	248	7867,32
	2	Case	36,22	42,25	53,09	129,42	14,73	31167,56	136,15	33,14	191	5524,36
	3	Case	37,91	47,07	60,82	149,18	19,40	41748,89	130,26	45,74	185	7133,45
	4	Case	32,99	40,65	44,97	123,72	10,79	41622,44	131,66	21,38	261	39732,40
53	1	Case	60,42	151,63	86,09	215,59	36,78	92391,84	196,65	3,70	349	6109,04
	2	Case	42,18	45,50	54,41	145,84	22,80	80181,32	131,66	24,56	316	21230,63
	3	Case	63,53	83,91	67,23	179,26	34,79	90098,39	153,21	< 1,5	257	19706,04
54	1	Case	25,90	48,18	34,20	85,10	6,36	29412,92	97,57	5,83	326	11245,39
	2	Case	35,38	52,26	47,46	129,42	19,40	35229,84	115,41	4,96	345	1348,31
	3	Case	42,12	53,56	54,50	149,18	19,40	49324,17	136,15	13,06	315	2594,86
	1	Case	25,59	42,35	40,28	119,97	1,28	24706,75	95,27	44,14	281	2699,46

56	2	Case	50,00	94,36	69,42	177,47	34,79	99414,91	146,89	21,54	201	10893,40
	3	Case	42,12	36,81	64,34	168,54	19,40	57026,42	144,93	< 1,5	286	4487,38
57	1	Case	23,28	43,39	38,16	89,32	15,67	35633,56	132,92	61,54	329	4776,33
	2	Case	36,22	84,04	64,34	173,32	19,40	52441,32	136,15	5,52	288	17224,81
58	1	Case	29,91	33,18	38,40	123,72	10,79	31771,89	158,43	63,78	300	16248,68
59	1	Case	50,43	190,10	53,46	142,20	18,94	72863,36	1529,22	215,00	219	20747,29
60	1	Case	43,57	77,32	58,75	157,86	18,48	68717,51	140,17	35,95	372	4089,91
	2	Case	224,32	709,07	122,55	192,27	23,90	55704,76	168,10	11,94	221	9700,41
61	1	Case	37,28	112,39	52,52	160,28	22,80	230790,81	149,55	41,21	174	18011,99
62	1	Case	42,12	26,12	51,97	124,89	21,69	42417,90	129,14	<1,5	187	1145,28
63	1	Case	32,84	68,08	67,15	168,54	19,40	54726,80	193,81	71,74	371	6670,67
	2	Case	21,25	32,46	44,97	118,09	14,95	41828,86	120,38	4,59		7502,51
	3	Case	34,14	104,92	65,59	154,06	31,52	53399,04	808,99	361,20	388	10312,61
64	1	Case	23,11	27,66	38,87	112,40	6,36	32180,78	106,74	26,06	272	2764,61
	2	Case	26,86	55,84	53,79	149,18	14,73	47734,81	118,40	11,14	191	5615,10
	3	Case	29,21	40,21	49,80	128,56	21,69	44076,47	108,77	10,53	173	4621,68
	4	Case	24,35	58,71	45,91	116,20	6,36	47495,91	99,87	10,53	173	14396,55
65	1	Case	42,18	36,55	54,41	142,20	16,96	82717,89	111,30	<1,5	169	31463,29
	2	Case	41,26	48,91	52,52	142,20	14,95	79868,85	120,38	3,86	132	14995,22
66	1	Case	47,68	56,01	61,05	179,78	22,80	122312,20	143,97	29,31		22764,15
	2	Case	62,49	128,28	82,58	215,59	23,90	105792,54	185,27	41,22	391	14157,58
	3	Case	60,42	474,31	88,19	220,21	32,56	111157,09	196,65	< 1,5	345	17112,36
67	1	Case	38,51	29,58	50,63	131,17	10,79	71491,25	104,45	2,88	1025	2797,59
	2	Case	36,67	42,83	47,80	116,20	10,79	48104,57	97,57	5,21	441	14476,06
	3	Case	31,45	34,14	44,97	112,40	8,62	41308,15	90,64	4,28		1258,58
68	1	Case	47,13	124,91	69,95	187,56	19,40	73740,03	147,85	<1,5	194	7196,02
69	1	Case	42,47	54,17	53,06	139,53	28,25	34070,65	134,22	50,84	316	2847,56
	2	Case	47,97	441,63	68,55	182,83	23,90	54431,38	142,01	27,95	430	NO FOUND
	3	Case	31,14	52,82	43,09	119,97	18,94	36967,31	122,64	83,55	370	9022,56
70	1	Case	29,91	29,34	43,09	119,97	10,79	41359,09	131,66	37,53	346	4242,57
	2	Case	54,62	115,67	76,97	206,31	23,90	97695,01	173,84	2,84	226	5977,39
71	1	Case	39,43	35,10	50,63	131,17	14,95	59090,21	122,64	6,87	232	13320,82
72	1	Case	30,53	30,78	47,80	123,72	6,36	25227,36	113,58	8,40	223	5277,95
	2	Case	42,12	44,50	65,74	163,73	23,90	33101,01	147,85	6,76	197	1590,32
	3	Case	34,83	37,27	59,15	167,41	14,95	40257,93	129,41	4,14	210	5251,55
73	1	Case	26,23	45,22	52,86	122,66	18,48	64638,76	123,08	5,94	185	7068,81
	2	Case	40,86	264,62	74,87	201,65	19,40	80065,18	170,97	33,72	258	12046,13
	3	Case	38,75	91,68	74,87	192,27	23,90	82174,26	168,10	9,59	168	3537,85
74	1	Case	40,44	126,03	55,90	168,54	19,40	65422,83	153,66	15,73	161	8616,58
	2	Case	40,02	221,47	75,57	192,27	28,28	78118,64	150,75	< 1,5		
75	1	Case	29,91	24,79	43,09	116,20	10,79	39653,37	113,58	19,01	406	3480,40
	2	Case	35,54	31,97	47,63	113,83	24,97	34945,54	136,76	16,46	300	6136,85
	3	Case	47,97	66,08	68,55	178,09	23,90	73959,93	156,55	<1,5	227	1863,68

	1	Case	31,76	35,59	43,09	116,20	10,79	31420,41	120,38	38,76	189	3539,35
76	2	Case	51,30	70,43	75,57	173,32	32,56	67299,98	178,13	26,53	190	6547,18
	3	Case	52,13	107,67	75,57	182,83	23,90	76476,44	159,45	3,10	199	5158,92
	1	Case	38,9	424,58	49,97	134,97	33,95	52346,56	124,94	8,23	223	
77	2	Case	40,40	502,89	58,50	154,06	34,79	50203,00	131,68	52,93	235	10931,05
	3	Case	45,85	396,87	64,85	200,68	46,27	41038,97	147,32	44,79	220	17780,40
78	1	Case								65,36	484	
79	1	Case	34,83	35,59	43,09	127,45	10,79	39812,00	106,74	7,98	210	5991,80
	2	Case	36,22	40,01	60,12	149,18	19,40	53099,52	190,97	< 1,5	178	1748,41
	1	Case	50,00	148,98	65,05	161,29	38,06	56600,43	159,53	98,75	323	22047,41
80	2	Case	55,43	115,25	67,23	164,89	39,69	80099,96	162,05	52,49	201	NO FOUND
	3	Case	66,89	858,41	83,69	189,99	44,57	136345,55	157,00	33,33	255	34241,36
81	1	Case	34,83	30,54	43,09	116,20	10,79	37794,43	153,99	27,27	364	10014,11
	2	Case	52,38	101,80	63,95	154,06	31,52	54896,46	182,19	22,00	289	11451,36
	1	Case	38,51	54,29	43,09	116,20	6,36	34876,15	165,07	62,09		32824,33
82	2	Case	35,44	55,02	50,16	142,20	6,36	52217,69	121,51	538,40		43263,61
	3	Case	36,27	56,34	53,96	146,24	6,42	52462,98	126,34	434,77		
83	1	Case	48,91	143,91	58,20	149,47	22,80	101510,96	256,47	188,00	446	56354,82
	2	Case	38,51	69,12	57,25	138,54	18,94	88375,78	131,66	24,60		6613,86
	1	Case	55,43	65,45	73,80	179,26	31,52	96694,48	188,48	21,37	358	14875,97
84	2	Case	36,98	89,56	50,63	138,54	14,95	62702,13	162,86	152,90	461	36065,06
	3	Case	40,96	49,89	56,30	163,85	14,95	130150,75	149,55	2,17	502	11557,55
85	1	Case	47,84	34,54	62,24	142,43	24,16	98625,27	142,57	134,10		51048,31
	2	Case	56,78	130,43	71,61	189,99	34,79	102035,00	151,95	134,10	420	16023,12
86	1	Case	52,72	54,75	56,32	148,62	21,69	38857,79	113,87	470,40	527	14969,67
	1	Case	24,76	22,68	31,68	75,99	12,89	29906,91	137,76	37,17	286	4446,32
87	2	Case	40,71	33,49	51,66	140,48	18,48	68375,13	222,13	683,60	414	19266,03
	3	Case	53,79	94,84	68,55	194,62	23,90	79613,38	196,65	6705,00	319	12564,62
	1	Case	45,21	93,13	48,72	121,21	21,69	34884,44	121,52	60,86	355	28413,00
88	2	Case	39,73	114,77	57,25	147,66	26,58	67213,06	122,64	6,29		38380,72
	3	Case	36,67	72,12	47,80	116,20	14,95	47897,42	114,71	5,27		58618,53
89	1	Case	33,29	53,06	46,85	127,45	10,79	54141,90	160,65	491,70	510	20682,58
	2	Case	38,51	50,37	53,46	138,54	10,79	84913,70	131,66	483,57	292	17731,33
	1	Case	53,79	88,89	86,79	215,59	23,90	103311,64	165,22	6,48	486	6262,87
90	2	Case	48,63	79,03	69,42	157,67	28,25	79881,34	148,16	19,46	327	5546,23
	3	Case									243	
	1	Case	39,38	52,43	59,42	160,34	24,64	45782,22	172,48	525,70	271	
91	2	Case	40,74	50,35	56,32	128,56	28,25	46836,01	151,95	442,50	400	3915,78
	3	Case									293	
	1	Case	43,16	83,91	53,60	132,22	24,97	44417,66	188,48	418,00	413	523,71815
92	2	Case	48,20	81,96	67,99	161,66	24,16	106891,13	154,41	175,20	325	7548,42945
	3	Case									229	
	1	Case	55,43	41,36	73,80	193,56	38,06	147691,46	155,74	59,48	556	15088,59

93	2	Case	43,84	99,32	53,06	128,56	24,97	44498,42	149,42	152,80	401	24618,01
	3	Case	45,90	75,08	56,86	124,89	28,25	49780,77	139,29	5,06	328	14311,46
94	1	Caso	32,99	23,36	52,52	134,86	10,79	60599,19	122,64	8,27		
	2	Caso	38,75	57,15	65,74	163,73	14,73	62215,98	156,55	3,69		
95	1	Caso	40,40	502,89	58,50	154,06	34,79	50203,00	131,68	64,22		

TABLE 7 CONTROL GROUP BASECYTOMETRY

CASE Nº	TNF-A	IL-8	IL-1B	IFN G	IL-17A	P SEL	G-CSF	IL6	Cf DNA
1	53,53	299,88	64,54	138,53	18,48	77570,23	135,34	19,29	3778,19
2	36,76	43,65	59,91	134,60	15,67	76635,09	148,51	6,51	5864,92
3	57,78	99,74	55,23	130,64	18,48	60299,90	118,08	5,10	1735,02
4	38,56	49,65	54,04	130,64	21,31	71508,79	132,92	insuficiente	8971,07
5	41,07	311,34	54,04	122,66	18,48	61021,71	123,08	10,56	3537,46
6	49,27	78,41	58,75	134,60	21,31	82205,50	141,37	7,05	3172,35
7	43,92	84,97	67,99	154,03	21,31	100133,44	130,47	5,41	4767,31
8	47,84	127,18	65,69	146,32	24,16	86833,56	134,13	8,89	2449,06
9	66,28	120,12	65,69	142,43	24,16	72539,98	152,06	5,98	1393,93
10	37,84	63,36	56,40	126,67	21,31	80151,77	128,02	21,55	3748,14
11	39,28	44,69	61,08	138,53	21,31	79103,99	140,17	10,30	3370,18
12	39,28	31,40	63,39	138,53	21,31	94021,12	144,95	3,60	1728,33
13	60,62	141,55	65,69	150,18	27,03	101088,11	140,17	10,62	1903,21
14	32,07	32,71	43,17	93,64	15,67	40052,00	106,58	7,83	621,45
15	42,85	175,18	65,69	142,43	21,31	80554,57	154,41	8,58	6362,27
16	42,14	40,53	52,86	134,60	21,31	67619,41	147,33	5,17	2410,52
17	39,99	46,00	51,66	114,55	18,48	63407,96	113,01	7,45	1694,89
18	32,80	28,52	58,75	130,64	29,92	74425,87	132,92	3,50	1487,56
19	42,85	52,01	59,33	126,67	24,16	77338,17	130,47	9,84	3162,93
20	42,50	149,17	62,24	142,43	24,16	80445,53	120,58	12,81	2561,00
21	39,99	57,27	59,91	134,60	27,03	79146,96	128,02	12,43	2499,35
22	32,07	50,18	38,16	110,45	18,48	24212,35	72,07	9,75	598,04
23	44,99	54,11	61,08	142,43	18,48	98235,18	132,92	9,53	1648,08
24	38,92	54,64	54,04	114,55	18,48	58902,43	130,47	8,06	2932,19
25	39,99	124,82	57,58	134,60	21,31	72320,80	110,45	13,16	1662,78
26	38,56	36,36	51,66	130,64	18,48	67487,89	128,02	6,36	2584,41
27	44,28	35,58	61,08	157,86	21,31	97898,21	119,33	11,10	1497,03
28	41,42	54,90	62,24	142,43	24,16	100173,05	154,41	3,80	3256,56
29	54,95	151,01	61,66	142,43	21,31	79556,21	130,47	6,64	4847,68
30	39,99	52,01	54,04	122,66	21,31	71135,11	118,08	7,20	3397,01
31	38,56	128,66	56,40	146,32	21,31	87312,10	144,95	11,33	832,13
32	40,35	84,42	61,66	148,25	21,31	79018,10	132,92	4,76	9230,71
33	38,56	47,56	59,91	136,57	18,48	80293,09	131,70	6,61	7014,88
34	41,42	38,45	56,40	130,64	21,31	74558,51	118,08	3,96	4251,55
35	42,14	42,87	62,24	138,53	18,48	89154,04	130,47	3,63	2898,75
36	32,07	29,31	50,47	118,62	18,48	61820,39	107,88	3,83	2370,39
37	96,80	226,36	72,54	154,03	29,92	83142,73	144,95	8,15	3685,46
38	39,99	83,05	52,26	130,64	15,67	63087,78	130,47	10,37	2857,07
39	36,76	50,44	58,75	270,74	18,48	77433,03	132,92	4,52	3524,09
40	41,07	37,14	57,58	132,62	21,31	80707,44	135,34	6,38	1234,61
41	44,99	56,21	59,91	154,03	21,31	81475,53	137,76	15,44	2487,43
42	46,42	229,82	71,40	138,53	21,31	87241,93	135,34	12,51	5356,63
43	36,04	25,88	51,66	114,55	18,48	55639,89	128,02	3,65	9635,51
44	41,78	87,73	62,81	142,43	24,16	96970,41	140,17	7,78	1330,39
45	34,24	49,92	49,27	118,62	18,48	61021,71	120,58	5,35	3704,67
46	31,35	28,78	46,85	102,13	18,48	56065,69	120,58	5,42	1812,31
47	42,85	93,56	61,08	142,43	27,03	95087,71	135,34	3,27	1778,49
48	52,82	71,91	71,40	152,11	21,31	98157,32	170,65	4,21	1002,47
49	57,07	115,75	61,66	130,64	21,31	70140,22	144,95	7,22	2351,14
50	34,96	28,78	50,47	114,55	14,27	63052,29	129,25	4,92	2189,42
51	38,56	31,14	54,64	130,64	21,31	66638,16	130,47	9,19	823,19

52	45,71	71,11	61,08	146,32	24,16	98066,56	137,76	9,14	2728,20
53	39,28	74,07	51,66	134,60	18,48	72340,70	128,02	11,17	1633,13
54	44,99	68,96	59,91	126,67	19,89	77443,58	114,28	10,21	1731,68
55	49,27	144,89	64,54	134,60	18,48	68755,64	125,56	16,42	2770,52
